# Neonatal GABAergic transmission primes vestibular gating of output for adult spatial navigation

**DOI:** 10.1007/s00018-024-05170-x

**Published:** 2024-03-19

**Authors:** Qiufen Jiang, Kenneth Lap-Kei Wu, Xiao-Qian Hu, Man-Him Cheung, Wenqiang Chen, Chun-Wai Ma, Daisy Kwok-Yan Shum, Ying-Shing Chan

**Affiliations:** 1 School of Biomedical Sciences, Li Ka Shing Faculty of Medicine, The University of Hong Kong, 21 Sassoon Road, Hong Kong, Hong Kong SAR People’s Republic of China; 2 State Key Laboratory of Brain and Cognitive Sciences, The University of Hong Kong, Hong Kong, Hong Kong SAR People’s Republic of China; 3 Present Address: F.M. Kirby Neurobiology Center, Boston Children’s Hospital, Harvard Medical School, Boston, MA USA; 4 Present Address: MRC Laboratory of Molecular Biology, Cambridge, UK; 5 Present Address: Department of Rehabilitation Medicine, The First Affiliated Hospital, Sun Yat-Sen University, Guangzhou, China; 6 Present Address: Joslin Diabetes Center, Harvard Medical School, Boston, MA USA

**Keywords:** Inhibitory gating, Development, Early GABAergic transmission, Vestibular input, Spatial navigation

## Abstract

GABAergic interneurons are poised with the capacity to shape circuit output via inhibitory gating. How early in the development of medial vestibular nucleus (MVN) are GABAergic neurons recruited for feedforward shaping of outputs to higher centers for spatial navigation? The role of early GABAergic transmission in assembling vestibular circuits for spatial navigation was explored by neonatal perturbation. Immunohistochemistry and confocal imaging were utilized to reveal the expression of parvalbumin (PV)-expressing MVN neurons and their perineuronal nets. Whole-cell patch-clamp recording, coupled with optogenetics, was conducted in vitro to examine the synaptic function of MVN circuitry. Chemogenetic targeting strategy was also employed in vivo to manipulate neuronal activity during navigational tests. We found in rats a neonatal critical period before postnatal day (P) 8 in which competitive antagonization of GABAergic transmission in the MVN retarded maturation of inhibitory neurotransmission, as evidenced by deranged developmental trajectory for excitation/inhibition ratio and an extended period of critical period-like plasticity in GABAergic transmission. Despite increased number of PV-expressing GABAergic interneurons in the MVN, optogenetic-coupled patch-clamp recording indicated null-recruitment of these neurons in tuning outputs along the ascending vestibular pathway. Such perturbation not only offset output dynamics of ascending MVN output neurons, but was further accompanied by impaired vestibular-dependent navigation in adulthood. The same perturbations were however non-consequential when applied after P8. Results highlight neonatal GABAergic transmission as key to establishing feedforward output dynamics to higher brain centers for spatial cognition and navigation.

**Supplementary Information:**

The online version contains supplementary material available at 10.1007/s00018-024-05170-x.

## Background

Head orientation is already detected by vestibular organs at birth [1] and registered by neonatal vestibular nucleus (VN) neurons that receive their inputs [2, 3], but multiple key molecular events need to occur within the early postnatal stage for establishment of a functional spatial cognitive system. In the rat VN, one such event is NMDAR-dependent unsilencing of neurons at P5–7 [4]. The excitatory drive needed for activation of NMDAR in the neonatal brain is known to be provided by early depolarizing γ-aminobutyric acid (GABA)-ergic transmission [5]. Timely unsilencing is critical for integration of vestibular outputs into ascending circuits for spatial navigation [4]. Competitive antagonization of GABA_A_ receptor (GABA_A_R) during the first postnatal week delayed emergence of graviceptive negative geotaxis reflex [6], indicating the importance of GABAergic transmission in normal development of nascent VN circuits.

Does perturbed GABAergic transmission in the early postnatal stage impact assembly of local inhibitory interneuron circuits within the VN to affect feedforward shaping of outputs to higher centers of spatial cognition? Head motion inputs processed by VN circuits are fed into the ascending vestibulo-thalamic pathway via the supragenual nucleus (SGN) [7–9]. This supports spatially correlated activity of thalamic head direction cells [10, 11], which are prerequisite for place cell and grid cell function [12, 13]. Together these hierarchical neuronal systems provide metric representations of space [14]. Two lines of evidence show that vestibular inputs serve as the basis for spatial cognitive circuits. First, head direction cells, operating mainly based on vestibular inputs, in rat pups before eye opening are already capable of spatially correlated activity [15, 16]. Furthermore, rodents with defunct vestibular input exhibit unstable activity in all levels of neural representations of space [17], despite normal visual input. As such, derangements in processing of vestibular inputs in the VN are likely to result in far-reaching consequences towards spatial cognitive function.

To reveal the effects of early GABAergic transmission on the assembly of VN circuits for sensory processing, we first identified a neonatal critical period in which blockade of GABAergic transmission caused long-lasting deficits in spatial navigational behavior. Such perturbation increased the number of GABAergic parvalbumin (PV)-expressing neurons in medial vestibular nucleus (MVN), which is the largest and most extensive VN subnuclei in connection to higher cognitive areas. But the ratio of excitatory and inhibitory transmission (E/I ratio) therein was also paradoxically increased. Though PV neurons are not the only GABAergic interneuron subtype within the MVN [18], we showed that PV neurons were indispensable for efficient navigation using chemogenetic inhibition. With neonatal antagonization of GABAergic transmission, input from PV-expressing interneurons to SGN-projecting MVN output neurons in adult rats was significantly reduced in the adult MVN. This suggested that PV-expressing interneurons in the MVN mediate inhibitory gating of the vestibular outputs along the ascending pathway for spatial cognition. Such change in circuitry distorted both spiking and intrinsic excitability of these SGN-projecting MVN output neurons. Our results thus highlight a role for neonatal GABAergic transmission in the postnatal assembly of inhibitory circuits at MVN output neurons that shape information flow to support spatial navigation.

## Materials and methods

### Animals

Sprague–Dawley (SD) rats (at P1, 6, 8, 9, 12, 14, 57 and 60), Pvalbtm1.1(cre)Aibs (PV-Cre) mice (Jackson Laboratory, JAX 008069) (at P1, 8, 42, and 60), and C57BL/6 J mice (at P1, 10, 60) of either sex were used (Supplementary Table 1). All animal procedures were conducted according to the Guide for the Care and Use of Laboratory Animals (National Institutes of Health) and approved by the University of Hong Kong Committee on the Use of Live Animals in Teaching and Research. The day of birth of each pup was recorded as P1 and the litters were reared with a lactating female. All animals were housed in the Centre for Comparative Medicine Research under a 12-h light/dark cycle with ad libitum access to food and water. Effort was made to keep the animals used at a minimum and to decrease animal suffering.

### Navigational behavior: dead reckoning test

The ability of rats and mice to return home via a direct path after foraging for food in an open field was assessed in the dead reckoning test [19, 20]. Rodents (both rats and mice) underwent behavioral acclimatization and baseline-cue training 1 week before conducting the dead reckoning test at P60. The test arena was a round table (205 cm in diameter for rats; 120 cm for mice) with 8 holes (11.5 cm in diameter each for rats; 5 cm for mice) arranged at equal distance around the perimeter of the table. These holes were designed to allow a plexiglass box (the “home base”) to be affixed underneath. The table was located 35 cm above the floor in a test room with multiple visual cues, including a ceiling-to-floor black curtain around the arena, computer boxes, a chair, and a refrigerator, which remained during the entire experimental period. Activities of the rodents on the table were recorded using a video camera with infrared taping abilities (HI-8 Sony) positioned above the centre of the round table.

Rodents were fasted for 12 h prior to training and test sessions and brought into the experimental room 1 h before the test began. Training differed slightly between rats and mice. For acclimatization, the rat was first placed on the table to forage for food pellets (1 g Supreme Mini-Treats pellets, Bio-Serv). Then, rats were subjected to baseline-cue training in which the rat was permitted to leave its home base (placed in the same location throughout the training session) through the hole to search for 4 randomly placed food pellets on the table within a 30 min time limit. Rats spontaneously carried each food pellet directly back to the home base. As mice would eat the pellet on the table, supervised training was required [19]. Only 1 pellet (45 mg Dustless Precision Pellets, Bio-Serv) was placed randomly on the table in each trial. When the mouse started to eat the pellet, the trainer would ring a bell and remove the pellet. Another pellet was placed on the table only after the mouse returned to its home base. Mice learned to carry the pellet back to the home base for consumption after 1 week of training. Up to 4 training sessions were given daily for all rodents. After each trial, the surface of table was rinsed completely with 75% ethanol so as to minimize olfactory cues. A trial was complete if the rodent (1) left the home base to search for food on the table, (2) found the food pellet, and (3) returned to the home base with the pellet. Rodents were considered ready for dead reckoning test when they completed 5 consecutive trials.

The dead reckoning test consisted of light probe test, dark probe test and new home location probe test. During each test session, 1 food pellet was placed randomly within a circular area (diameter 30 cm for rats, 20 cm for mice) around the middle of the table. The animal used both self-movement cues and environmental cues in the light probe test during which the lights in the room were kept on.

The dark probe test was conducted in the dark together with a ceiling-to-floor black curtain completely surrounding the table so as to remove visual cues. Rodents navigated mainly using self-movement cues. The dark and light test probes were carried out on alternate days. Four test sessions were conducted per day for each rodent. At least 8 successful test sessions in both light and dark probe tests were recorded and analyzed for each rodent.

In the new home location test, animals were released from a home base that was diametrically opposite to the original home base used for light and dark probe tests. Animals were subjected to 2 trials per day for 2 consecutive days. Completing the new location test required the self-movement cues generated during the outward-bound path of the animals since this test introduced a conflict between the allothetic cues acquired during baseline-cue training.

The paths of rodents were analyzed offline using TopScan (CleverSys Inc, USA) by experimenters blinded to the pre-treatment of the animals. Parameters measured include the duration of searching time and returning time (s), the heading angle (deg), and the mistakes undertaken during returning (number of errors).

### Chemogenetic inhibition of PV-expressing interneurons during navigational behavior

Clozapine-N-oxide (CNO, Cayman, 16882) was dissolved in physiological saline to a working concentration of 0.1 mg/ml. To chemogenetically inhibit PV-expressing interneurons in the VN, CNO (1 mg/kg) was intraperitoneally administrated 30 min prior to dead reckoning test to mice that had been pre-transfected with virus carrying expression vector for hM4DGi (AAV5-hSyn-DIO-hM4D_Gi_-mCherry) and received baseline-cue training as described above. To minimize the confounding effect of CNO metabolite, control animals were administered with CNO (1 mg/kg) as comparison.

### Preparation and implantation of bicuculline-loaded Elvax slice

Biopolymer Elvax 40P (Dupont, 1 g) was dissolved in 10 ml of dichloromethane (10%, w/v, Merck) at room temperature (22–24 °C). Saline (lyophilized with Ficol, Sigma) or GABA_A_R antagonist bicuculline (BIC, 20 mM, Tocris, 0109) was mixed with 200 µl of dichloromethane and then added to the dissolved Elvax. The mixture was vortexed before frozen in liquid nitrogen and then stored at − 80 °C overnight. The Elvax block was kept at − 20 °C for 2 weeks before sectioning into 200 µm thick slices. To confine the direction of drug release from the Elvax slice only from one surface and not the other, one side of the Elvax slice was coated with polycaprolactone (PCL) (Sigma) using a spray gun [21]. With the use of ^3^H-BIC-loaded Elvax slices (1 mm × 1 mm, 200 µm thickness, 20 mM ^3^H-BIC), we have demonstrated PCL coating on both sides retained 95% of the loaded drug within the slice even after immersing in PBS for 1 week, while uncoated Elvax slices released the majority of the drug in the same time period [6].

Under isoflurane anesthesia (5%, 250 cm^3^/min, Zoetis), the fourth ventricle of P1 rats or mice was surgically exposed. A piece of Elvax slice (200 µm thick, 1 mm × 1 mm for rats, 0.8 mm × 0.8 mm for mice) loaded with saline or BIC was inserted into the fourth ventricle and placed on the surface of the bilateral VN such that the PCL-coated side of the Elvax slice faced the cerebellar primordium. Particular care was taken during this implantation not to damage the surrounding brain tissues. The skin incision was subsequently sutured. Pups were allowed to recover inside an animal intensive care unit (Thermocare) set to 35 °C before returning to their mothers. Their pups were given neomycin ointment, oral meloxicam and subcutaneous buprenorphine injection for 7 days post-operation. These pups showed positive health status and were kept until they were subjected to different behavioral, electrophysiological or immunohistochemical experiments. Animals were only included for analysis when at the end of the experiment both the Elvax slice was still in situ on the surface of the bilateral VN and the posterior cerebellum was intact.

### Viral vectors

Viral vectors were injected to express ChR2 or designer receptor exclusively activated by designer drugs (DREADD) in PV-expressing neurons. Recombinant adeno-associated viruses (AAVs) carrying Cre-activated expression vectors encoding either optogenetic activator ChR2 (AAV5-hSyn-ChR2-eYFP, Penn Vector Core) or the inhibitory DREADD hM4D_Gi_ (AAV5-hSyn-DIO-hM4D_Gi_-mCherry, Addgene) were purchased. Viral vectors were stored in aliquots at − 80 °C until use.

### Stereotaxic surgery and injection

For experiments employing both retrograde tracing and optogenetics, P42 PV-Cre mice pre-implanted at P1 with either saline- or BIC-loaded Elvax slice were anesthetized with isoflurane (5% induction, 2% maintenance, 250 cm^3^/min) and mounted in a stereotaxic apparatus (Stoelting Instruments). A 5 µl microsyringe (Hamilton) fitted with a glass capillary (tip diameter 15–20 µm) was slowly advanced through an opening on the skull dorsoventrally to the specific brain area. For retrograde tracing, red retrobeads (Lumafluor; 0.3 µl) was injected into the bilateral SGN of P57 rats (AP: − 10.5; ML: ± 0.6; DV: − 5.4 mm) or cholera toxin subunit B (CTB, Invitrogen; 0.3 µl) to the right SGN of mice (AP: − 5.3; ML: 0.25; DV: − 3.5 mm). AAV5-hSyn-ChR2-eYFP virus (Penn Vector Core; 0.3 µl) was delivered into the right MVN of mice (AP: − 6.2; ML: 0.5; DV: − 3.5 mm). Injections were made under the control of a micropump (World Precision Instruments) at 100 nl/min. Leakage from the injection site was minimized by slowly withdrawing the syringe after a delay of at least 10 min after each injection. The viral titers were > 2 × 10^13^ virus particles/ml and full transgene expression occurred after 2 weeks. The skin incision was subsequently sutured. Animals remained in a temperature-controlled intensive care unit (Thermocare) until fully recovered from anesthesia. These animals were given neomycin ointment, subcutaneous buprenorphine injection and oral meloxicam for 7 days post-operation. The animals were allowed to recover for 1–2 weeks before the in vitro electrophysiological experiments.

For chemogenetic experiments, P42 PV-Cre mice were anesthetized with isoflurane (5% induction, 2% maintenance, 250 cm^3^/min). AAV5-hSyn-DIO-hM4D_Gi_-mCherry was stereotaxically injected into the MVN on both sides of the brainstem as described above. The mice were allowed to recover for 2 weeks before behavioral experiments. After the dead reckoning test, immunohistochemical verification of mCherry in PV-expressing neurons within the MVN was done.

### Immunohistochemistry

Immunostaining was performed on brain sections from rats and mice. Rats pre-implanted at P1 with either saline- or BIC-loaded Elvax slice were sacrificed at P6, P9 or P12 with overdose of pentobarbital sodium (Dorminal, AlfaMedic International). Similarly, adult mice for co-staining of small conductance calcium-activated potassium (SK) channels and GABA_A_R were sacrificed. After transcardiac perfusion with 0.9% sodium chloride solution followed by ice-cold 4% paraformaldehyde in 0.1 M phosphate-buffered saline (PBS), the brain was carefully removed and post-fixed at room temperature for 4 h. The brain was washed with PBS and then transferred to ethanol:saline solution for 45 min and stored overnight in 70% ethanol, followed by dehydration through ethanol series (80, 90, 95%, and absolute ethanol for 30 min each). After dehydration, the brain was placed in fresh xylene twice for 10 min each and then equilibrated with wax in a vacuum oven overnight. After embedding in wax, coronal brainstem sections (7 µm) were obtained from the wax block using semi-automated microtome (HM340E, Microm International) and mounted on glass slides.

Before staining, the slices were deparaffinized in fresh xylene 3 times for 3 min each, following rehydration (100, 95, 70, 50, and 30% ethanol for 3 min each). After rinsing with PBS, antigen retrieval was conducted by bathing the specimen slides in boiled sodium citrate buffer (10 mM sodium citrate, 0.05% Tween 20, pH 6.0) for 20 min in a pre-heated steamer. The buffer dish with slides was moved to room temperature and allowed to cool for 20 min before slides were removed and rinsed twice with PBS.

Double staining of perineuronal nets (PNN) and PV in rat brain sections was performed with the incubation of biotinylated Wisteria floribunda agglutinin (1:200, WFA, Sigma, L-1516) and antibody against PV (1:1000; Abcam, ab11427). For comparing expression of SK channels with GABA_A_R in mice brain sections, antibodies against SK channels (KCNN2, 1:500; Alomone Labs, APC-028), GABA_A_R (1:500; Millipore, MAB341) and VGluT1 (1:500; Synaptic Systems, 135 304) were used. Slices were incubated in PBS with 3% normal goat serum and 0.3% Triton X-100 for immunofluorescent staining. Signals were visualized with TRITC-conjugated streptavidin or Alexa Fluor 488-conjugated secondary antibody against rabbit IgG. The slides were then washed and mounted. Images were taken using a fluorescent microscope (Olympus) with the same settings for each group. Further offline processing (Adobe Photoshop) was limited to brightness and contrast adjustments which were applied equally across the entire image and also applied equally to controls. The number of MVN cells immuno-positive to PV, WFA or both was counted from coronal slices, using ImageJ (NIH). The results from the same group were pooled for statistical analyses. Cell counting was conducted by individuals blinded to the experimental group.

### Brain tissue preparation for electrophysiology

Rats or mice of the desired age were decapitated under isoflurane anesthesia (5%, 250 cm^3^/min). For animals ≤ P14, the brains were immediately removed to ice-cold artificial cerebrospinal fluid (ACSF) composed of (in mM) 120 NaCl, 2 KCl, 2.5 CaCl_2_, 1.2 MgCl_2_, 1.2 KH_2_PO_4_, 26 NaHCO_3_, and 11 glucose, saturated with 95% O_2_ and 5% CO_2_ (adjusted to pH 7.3, 290 mOsm). Coronal brainstem slices containing VN (300 µm thick) were prepared on a vibratome (Campden Instruments, MA752) and then transferred to ACSF at 33 °C for 1 h [22]. The slices were then kept at room temperature with sustained aeration before recording.

For P60 animals, the brains upon removal from the skull were put into ice-cold N-Methyl-D-glucamine (NMDG; Sigma, M2004)-containing ACSF [23], composed of (in mM): 92 NMDG, 2.5 KCl, 1.25 NaH_2_PO_4_, 30 NaHCO_3_, 20 HEPES (Sigma; H4034), 25 glucose, 2 thiourea (Sigma; T7875), 5 Na-ascorbate (Sigma; A7631), 3 Na-pyruvate (Sigma; P2256), 0.5 CaCl_2_, and 10 MgSO_4_ × 7H_2_O (adjusted to pH 7.3, 290 mOsm). Coronal brainstem slices (250 μm) were cut on a vibratome. The slices were transferred to NMDG-ACSF at 33 °C for 10 min, and then placed in HEPES-ACSF consisting of (in mM): 92 NaCl, 2.5 KCl, 1.25 NaH_2_PO_4_, 30 NaHCO_3_, 20 HEPES, 25 glucose, 2 thiourea, 5 Na-ascorbate, 3 Na-pyruvate, 2 CaCl_2_, and 2 MgSO_4_ × 7H_2_O, at room temperature until recording.

Slices were transferred to a recording chamber mounted under an upright microscope equipped with infrared-differential interference contrast optics (BX51WI, Olympus) and a CCD camera. During recording, slices were perfused with ACSF containing (in mM) 119 NaCl, 2.5 KCl, 2 CaCl_2_, 1.25 NaH_2_PO_4_, 24 NaHCO_3_, 12.5 glucose, and 2 MgSO_4_ × 7H_2_O warmed to 32 °C using an inline heater (Warner Instrument Company) and aerated by bubbling with 95% O_2_ and 5% CO_2_ at a rate of 1.5 ml/min.

### Whole-cell patch-clamp recording

MVN neurons identified visually by location, size and fluorescence were recorded using borosilicate glass pipettes of external diameter 1.2 mm/internal diameter 0.69 mm (Harvard Apparatus) pulled from Flaming/Brown micropipette puller (Model P-97, Sutter Instrument) and filled with high chloride internal solution (in mM): 140 CsCl, 10 HEPES, 1 EGTA, 2 MgCl_2_, 2 Na_2_ATP, and 1 Na_2_GTP (adjusted to pH 7.2, 290 mOsm). The advancement of the pipette was manually operated through the micromanipulator (Sutter Instrument).

Signals were amplified using MultiClamp700A (Axon Instruments) and acquired through a 16-bit data acquisition system (DIGIDATA 1322A; Axon Instruments). During whole-cell patch-clamp recording, membrane potentials were corrected for the liquid junction potential (10 mV), and the change of series resistance was sustained within 15%. Only recordings with series resistance smaller than 15 MΩ were included for subsequent analysis. Cell recording was discarded if the leaking currents went beyond 200 pA. The signals of the recording were digitized at 10 kHz and filtered at 3 kHz by the Multiclamp 700A amplifier, Digidata 1322A analog/digital interface board and pCLAMP 10.2 software (Axon Instruments). Data were captured by Clampex 10.2/Multiclamp Commander 1 (Axon Instruments) package.

### Neuronal electrical properties/Spontaneous postsynaptic currents

sEPSCs of MVN neurons were recorded at − 70 mV with perfusion of ACSF containing BIC (10 μM) and antagonist of glycine receptor strychnine (Sigma; 1 μM) to remove inhibitory currents. Alternatively, sIPSCs were recorded at − 70 mV and isolated with bath application of AMPAR antagonist CNQX (Tocris; 10 μM) and NMDAR antagonist D-APV (Tocris; 50 μM).

SGN-projecting neurons in the MVN with red retrobeads were visualized under the fluorescence microscope. To activate vestibular afferent input, a bipolar tungsten electrode (225 µm tip separation, Microprobes) was placed in at the medial border between the lateral and medial vestibular nuclei to stimulate primary vestibular afferents. The stimulating electrode was connected to a current isolator (Grass Instrument). For experiments assessing synaptic plasticity, pulses of 0.1 ms duration were delivered at 0.05 Hz with stimulation intensity adjusted for every neuron such that the response was approximately midway between the minimal current and the saturated current (100–200 μA). Theta burst stimulation (TBS) consisting of 4 trains of 10 bursts delivered at 5 Hz with each burst containing 4 pulses at 100 Hz was employed to induce long-term plastic changes at GABAergic synapses. Neurons with a decrease in ePSC_GABA_ amplitude > 20% were categorized as exhibiting LTD_GABA_. GABA_A_R-mediated current was recorded in the presence of strychinine (1 μM). BIC was added to the bath after LTD measurements to confirm the GABA_A_R-mediated identity of the recorded current. Blue laser light (Lumen Dynamics) was used to opto-activate PV-expressing MVN interneurons, transduced with ChR2, through wide-field blue light illumination (473 nm, 1 min) via an optic fiber lens directed at the MVN region. To limit variability of photo-induced activation due to differences in viral vector expression, recordings were only made from slices in which ChR2-eYFP expression was visible at 4X magnification.

For recording of voltage responses, cells were injected with depolarizing current steps from 0 to 800 pA with incremental steps of 20 pA (each sweep lasted 1000 ms duration). The rheobase was defined as the minimal current that could elicit an action potential. Neurons which only responded with one action potential within the stimulation period were classified as single-spiking neurons, while those with more than 1 spike were classified as multiple-spiking neurons. Resting membrane potential was recorded in current clamp mode within the first minute of membrane rupture.

### Statistical analysis

No statistical methods were used to predetermine sample sizes, but our sample sizes were based on prior literature and best practices in the field. Data are presented as mean ± SEM. Graphpad Prism 8 was used to plot data and perform statistical analyses. Differences were considered statistically significance for *P* < 0.05. Data were subjected to D’Agostino-Pearson omnibus test for normal distribution and Levene’s test for equality of variances. Two-way ANOVA was used for Figs. 2A_2,3_, B_2,3_, 3F, and 5B. Paired student T-tests were used for data in Fig. 4C_3_, D_2_. For Figs. 4A_2_, B_2_, 6F_1,2_, G_1,2_, one-way ANOVA with Holm-Sidak’s multiple comparisons test was used for data with normal distribution. In cases when the data were not normally distributed, Dunn’s multiple comparisons test was used.

## Results

### Neonatal delivery of bicuculline to the vestibular nucleus deranges navigational behavior in adult rats

To investigate whether GABA signaling in the postnatal VN impacts on the acquisition of spatial navigation, we implanted an Elvax slice loaded with GABA_A_R antagonist BIC in the fourth ventricle over the VN at different postnatal stages (P1, P8 or P14). Navigational performance was then assessed by dead reckoning tests when these rats reached adulthood (P60) to reveal long-lasting changes to spatial navigational behaviour.

**Fig. 1:**
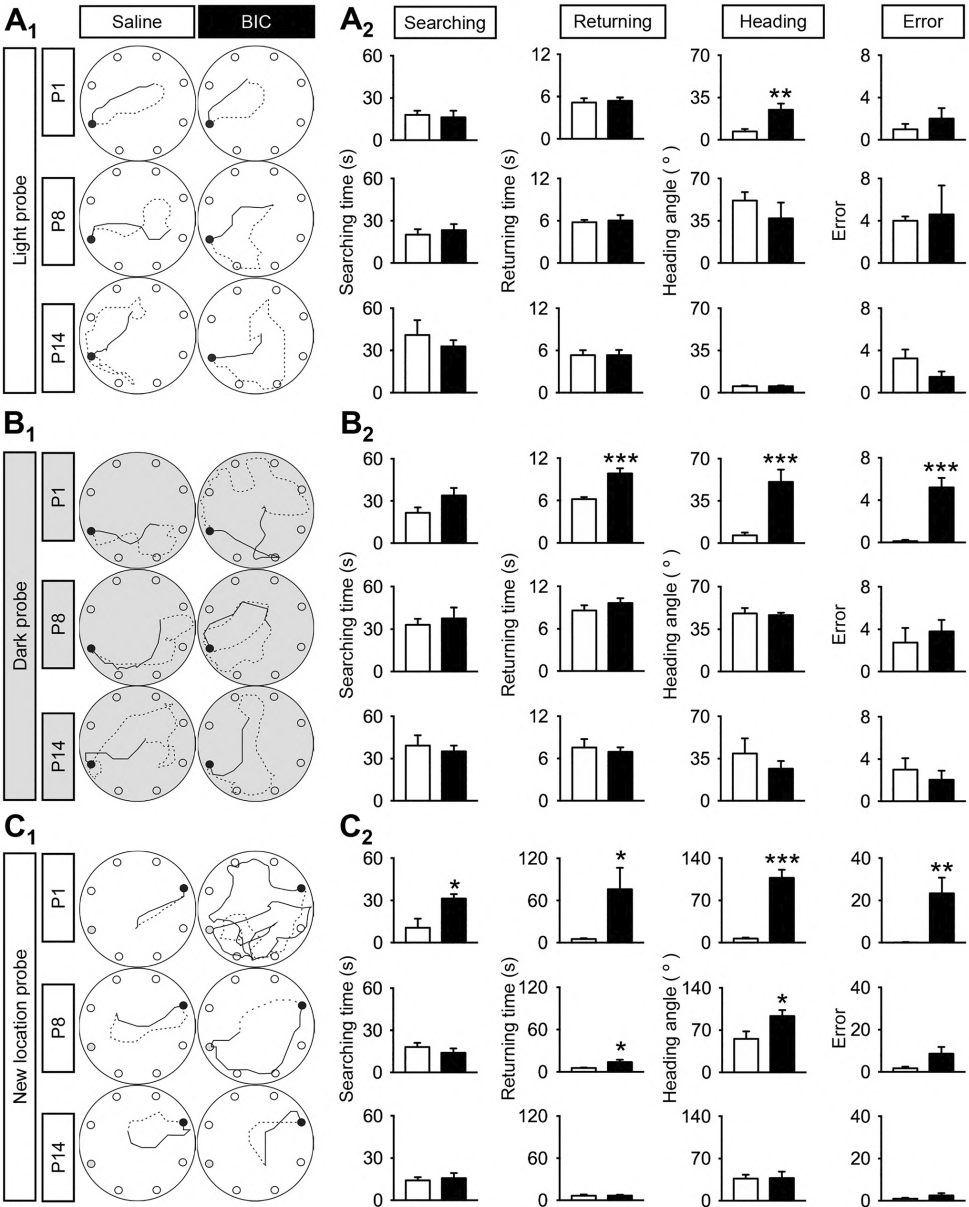
Neonatal GABAergic blockade deranges adult navigation performance. **A**_**1**_, **B**_**1**_, **C**_**1**_ Representative searching (dashed line) and returning (solid line) paths of adult (P60) rats pre-treated with saline or with BIC at P1 (upper panel), P8 (middle panel) or P14 (lower panel) under **A**_**1**_ light, **B**_**1**_ dark or **C**_**1**_ new location probes. Filled black circles indicate the location of home base. **A**_**2**_, **B**_**2**_, **C**_**2**_ Graph showing the average searching time (first column), returning time (second column), heading angle (third column), and errors in locating the home base (fourth column) of these rats. Data are presented as mean ± SEM. *P < 0.05, **P < 0.01, ***P < 0.001 indicate significant differences between the saline- and BIC-treated groups, independent samples t-test

When allothetic cues are available during the light probe test, animals preferentially navigate for food and return home using visual cues (Valerio and Taube, 2012). Adult rats pre-treated with BIC at P1 exhibited no significant difference in terms of their searching time (18.0 ± 2.9 vs. 16.2 ± 4.7 s; *P* > 0.05), returning time (5.2 ± 0.6 vs. 5.4 ± 0.5 s; *P* > 0.05), or number of mistakes in reaching home base (1.0 ± 0.5 vs. 2.0° ± 1.0°; *P* > 0.05) as compared to saline controls. However, these rats showed error in determining the direction of the homebase when they turned around to start the return trip after finding the food pellet (heading angle: 6.9 ± 1.9 vs. 25.0° ± 5.0°; *P* < 0.01) (Fig. 1A_2_). This suggested that the ability of integrating heading direction in these adult rats were retarded by localized BIC treatment to the VN at P1. Such deficits were compensated once the rats were in motion, perhaps due to additional heading information obtained from retinal flow. By P8 or P14, BIC-treatment to the VN could no longer affect the dead reckoning test performance in light probe condition of adult rats (Fig. 1A).

To dissociate light cues from the potential mechanisms of compensating heading direction in motion, we next performed dead reckoning test during the dark probe test. In complete darkness, self-movement cues, in particular vestibular information, are indispensable for efficient dead reckoning [20, 24]. Adult rats pre-treated with BIC at P1 showed significant deficits in assuming the correct heading direction (50.8 ± 10.23 vs. 6.44° ± 2.33° in controls; *P* < 0.001). As compared to those under light probe test, these rats took much longer time to return home (9.83 ± 0.73 vs. 6.21 ± 0.30 s in controls; *P* < 0.001) and made more mistakes on their way back (5.2 ± 0.92 vs. 0.13 ± 0.13 s in controls; *P* < 0.001) (Fig. 1B). However, no effect was observed with BIC treatment either at P8 or P14 (Fig. 1B). This reveals a critical period for GABAergic transmission to guide hardwiring of VN circuits for spatial navigation.

In the new home location probe test, both environmental and self-movement cues were employed by the animals to locate food pellets and new home base. Adult rats, with VN circuits disturbed by BIC treatment at P1, were disabled in seeking food pellets (31.4 ± 3.15 vs. 10.64 ± 6.43 s in controls; *P* < 0.001) and locating new position of the home base (76.11 ± 30.41 vs. 5.18 ± 1.19 s in controls; *P* < 0.05) (Fig. 1C). These rats also showed a much larger heading angle deviation from new home (107.50 ± 13.38 vs. 6.81° ± 1.59° in controls; *P* < 0.001) and made more errors on their way back (23.40 ± 7.37 vs. 0.13 ± 0.13 in controls; *P* < 0.01) (Fig. 1C). Rats pre-treated with BIC at P8 did not show any difficulty in searching food as compared with control rats (18.19 ± 2.91 vs. 14.05 ± 3.00 s; *P* > 0.05). These rats, however, took significantly more time to find the new home (5.69 ± 0.31 vs. 13.8 ± 3.5 s; *P* < 0.05), along with a larger heading angle deviation compared to controls (93.49 ± 9.87 vs. 55.56° ± 12.52°; *P* < 0.05) (Fig. 1C). BIC treatment after P14 had no effect on adult navigation ability (Fig. 1C).

These results show that early perturbation of GABAergic transmission in the VN deranged navigation performance in adulthood. Notably, the time point when VN circuits became insensitive to pharmacological perturbation was postponed when the navigation task was more dependent on higher order spatial cognitive functions such as spatial memory [25].

### Postsynaptic currents of MVN neurons in postnatal rats that were pre-treated with BIC at P1

We next investigated how neonatal inhibition of GABAergic transmission in the VN alters the local circuitry during development and how the disorganized circuits finally affect VN output signals for spatial navigation. To demonstrate neurodevelopmental basis for the observed behavioral deficits, the developmental trajectory of the excitation/inhibition (E/I) ratio in rats pre-treated with BIC was compared with age-matched controls.

**Fig. 2:**
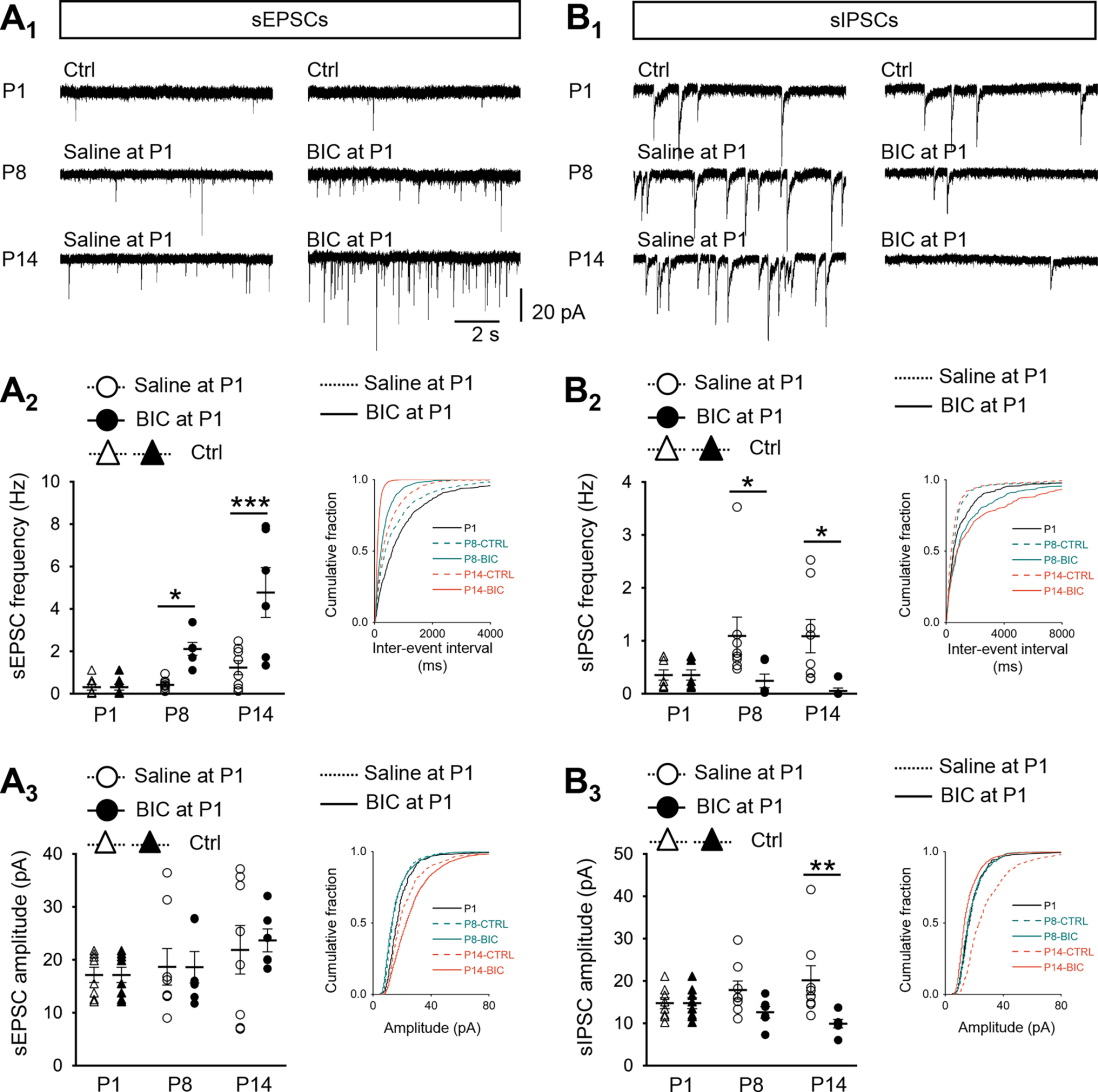
EPSC and IPSC profiles require neonatal GABAergic transmission for maturation. **A**, **B** sEPSCs were recorded in the presence of GABA_A_R antagonist BIC (10 μM) and glycine receptor antagonist strychnine (1 μM), while sIPSCs were recorded in the presence of AMPAR antagonist CNQX (10 μM) and NMDAR antagonist D-APV (50 μM). Representative traces showing sEPSCs (**A**_**1**_) and sIPSCs (**B**_**1**_) from P1 control rats (i.e. without surgery), P8 and P14 rats pre-treated with saline or BIC to the VN at P1. Charts illustrating the developmental changes in frequency (**A**_**2**_, **B**_**2**_) and amplitude (**A**_**3**_, **B**_**3**_) of sEPSCs or sIPSCs in P1 control rats (triangle and solid line), or P8 and P14 rats pre-treated at P1 with saline (open circle and dashed line) or BIC (filled circle and solid line). Insets show cumulative fraction of IEI or amplitude in postnatal rats with P1 pre-treatment of saline or BIC. *P < 0.05, **P < 0.01, ***P < 0.001, two-way ANOVA. Data are presented as mean ± SEM. Only significant differences between the saline- and BIC-treated groups are labeled. Fold changes in sPSC profiles after BIC treatment at P1 were showed in Supplementary Fig. 1. Detailed comparisons are shown in Supplementary Table 3

sEPSCs were recorded with the addition of GABA_A_R antagonist BIC and glycine receptor antagonist strychnine in the bath solution to remove inhibitory currents. In control rats, low frequency sEPSCs were detectable from MVN neurons as early as P1 (0.32 ± 0.14 Hz). Frequency of sEPSCs from MVN neurons showed a progressive increase from P8 (0.42 ± 0.09 Hz) to P14 (1.24 ± 0.33 Hz), although no statistical significance was detected (Fig. 2A_1,2_). Pre-treatment of BIC at P1 resulted in a significant increase in the frequency of sEPSCs at both P8 (2.11 ± 0.3 Hz; *P* < 0.001) and P14 (4.78 ± 1.18 Hz; *P* < 0.05), when compared to saline controls (Fig. 2A_1,2_). Accordingly, sEPSC frequency was significantly increased with age (*P* < 0.001). This is also reflected by the accumulation of shorter inter-event intervals (IEIs) in the probability curves of rats pre-treated with BIC (Fig. 2A_1,2_). In contrast, sEPSC amplitude did not show any significant change over development either in saline controls or BIC-treated rats (Fig. 2A_1,3_). The amplitudes between these two groups also showed no difference at any of the investigated ages (Fig. 2A_1,3_).

To remove sEPSCs, sIPSCs were recorded in the presence of AMPAR antagonist CNQX and NMDAR antagonist D-APV in the bath solution. In P1 control rats, the frequency of sIPSCs was 0.36 ± 0.1 Hz. For saline control rats, no statistical significance was detected in frequency of sIPSCs over development from P8 (1.09 ± 0.36 Hz) to P14 (1.09 ± 0.31 Hz) (Fig. 2B_1,2_). On the contrary, the frequency of sIPSCs in MVN neurons of BIC-treated rats declined to 0.24 ± 0.13 Hz at P8, further decreasing to a level that was hardly detectable at P14 (0.06 ± 0.01 Hz; *P* < 0.05, Fig. 2B_1,2_). Notably, there was a significant decline in the amplitude of sIPSCs in P14 rats that were pre-treated with BIC at P1 (9.95 ± 0.01 pA) when compared with saline controls (20.19 ± 3.4 pA;* P* < 0.05), while no significant change was observed in the P8 groups (Fig. 2B_1,3_). Both control and BIC-treated rats showed no significant change in sIPSCs amplitude over development from P1 to P14. These results are in line with the cumulative fraction curves of sIPSCs amplitude in the control and BIC-treated groups at P1, P8 and P14 (Fig. 2B_3_), indicating that lack of early GABAergic transmission deranged normal development of local circuits within the MVN.

To gain a better comparison in the change of excitatory and inhibitory transmissions over development, fold-change for both sEPSCs and sIPSCs was calculated by taking the ratio of the averaged frequency of BIC-treated rats to that of the controls (Supplementary Fig. 1A). The respective ratios for averaged amplitude (Supplementary Fig. 1B) were similarly calculated. The ratio of sEPSC frequency increased from 1.00 at P1 to 5.03 at P8, while slightly decreased to 3.87 at P14. On the contrary, the ratio of sIPSC frequency gradually declined to 0.22 at P8 and 0.05 at P14 (Supplementary Fig. 1A). As for the amplitude, sEPSC ratio did not show obvious changes during postnatal development, while sIPSC ratio exhibited a progressive decrease from P1 to P14 (Supplementary Fig. 1B). This further illustrates that BIC treatment at P1 increased excitatory transmission and reduced inhibitory transmission in the VN circuitry during the first 2 weeks of postnatal development. What then would be the long-term consequence of decreased inhibition in the developing MVN circuits?

### Long-term plasticity of MVN neurons in postnatal rats with pre-treatment of BIC at P1

**Fig. 3:**
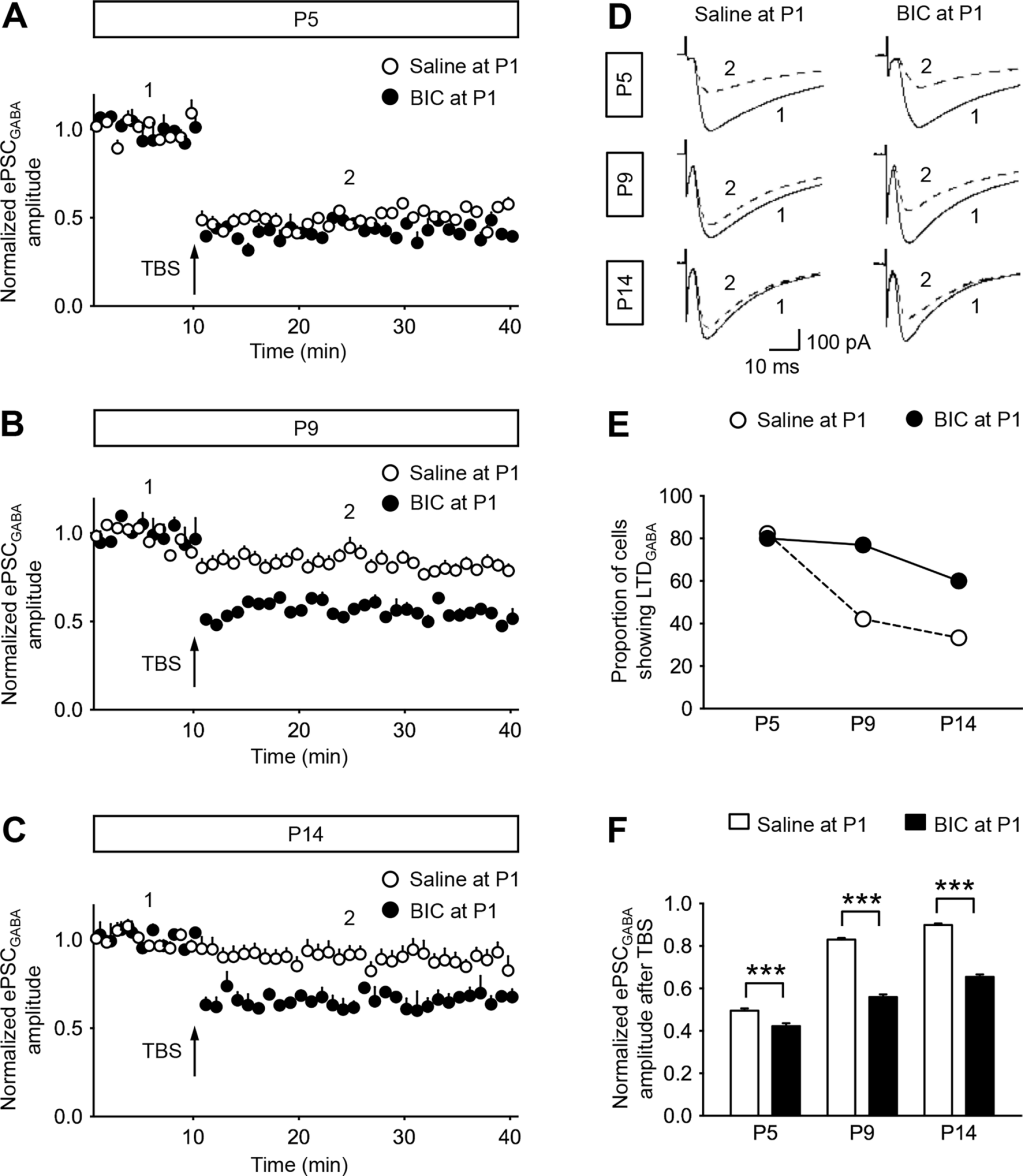
Suppression of neonatal GABAergic transmission extends postnatal period of high long-term plasticity mediated by GABA_A_R. **A**–**C** Normalized amplitudes of ePSC_GABA_ against recording time showing GABA_A_R-mediated long-term plasticity in P5 (**A**), P9 (**B**), and P14 (**C**) rats that received pre-treatment with saline (open circle) or BIC (filled circle) at P1. ePSC_GABA_ was isolated in the presence of AMPAR antagonist CNQX (10 μM), NMDAR antagonist D-APV (50 μM), and glycine receptor antagonist strychnine (1 μM). TBS was applied after 10 min of baseline recording. **D** Representative tracings of ePSC_GABA_ obtained before (1, solid line) and after (2, dashed line) TBS stimulation from P5, P9 and P14 rats that were pre-treated with saline or BIC at P1. **E** The proportion of cells exhibiting LTD_GABA_ in P9 and P14 rats pre-treated with BIC at P1 (filled circle) was increased compared to those pre-treated with saline at P1 (open circle). **F** Histogram showing a decrease in normalized amplitudes of ePSC_GABA_ after TBS in P5, P9 and P14 rats that were pre-treated with BIC (filled column) at P1 when compared with those pre-treated with saline at P1 (open column). ***P < 0.001, two-way ANOVA. Data are presented as mean ± SEM

It is known that the level of inhibition is inversely correlated with the degree of plasticity in cortical circuits, demonstrating the ability of neural networks to change through external experiences [26]. To quantify neuronal plasticity, we measured the percentage of neurons displaying TBS-induced long-term depression (LTD_GABA_) of evoked GABAergic postsynaptic currents (ePSC_GABA_) in the MVN of early postnatal rats. In P5 control rats, 82.35% of cells (n = 14/17 cells, 5 rats) exhibited LTD_GABA_ (Fig. 3A, E). This proportion decreased with age, reaching 42.11% at P9 (n = 8/19 cells, 6 rats) and 33.33% at P14 (n = 7/21 cells, 6 rats) (Fig. 3E). The normalized amplitudes of ePSC_GABA_ after TBS when compared to corresponding baselines before TBS showed a progressive increase from P5 to P14 (Fig. 3A–D, F; *P* < 0.001), indicating a decrease in the magnitude of LTD_GABA_.

For rats pre-treated with BIC at P1, the proportion of MVN cells exhibiting LTD_GABA_ remained stable between P5 (80%, n = 8/10 cells, 3 rats) and P9 (76.92%, n = 10/13 cells, 4 rats), but dropped to 60% at P14 (n = 6/10, 3 rats) (Fig. 3E). MVN neurons of these BIC-treated rats therefore exhibited a higher occurrence of LTD_GABA_ when compared with the saline controls. The normalized amplitudes of ePSC_GABA_ among MVN neurons of these BIC-treated rats also exhibited an age-dependent increase from P5 to P14 (*P* < 0.001). Furthermore, the normalized amplitudes of such ePSC_GABA_ were larger in BIC-treated rats than the controls (Fig. 3A–D, F; *P* < 0.001). These showed that synaptic strength at GABAergic synapses within the MVN was more readily attenuated after neonatal BIC treatment, leading to a decrease in the overall level of inhibition within the MVN throughout postnatal development.

### Role of PV-expressing interneurons in spatial navigation

**Fig. 4:**
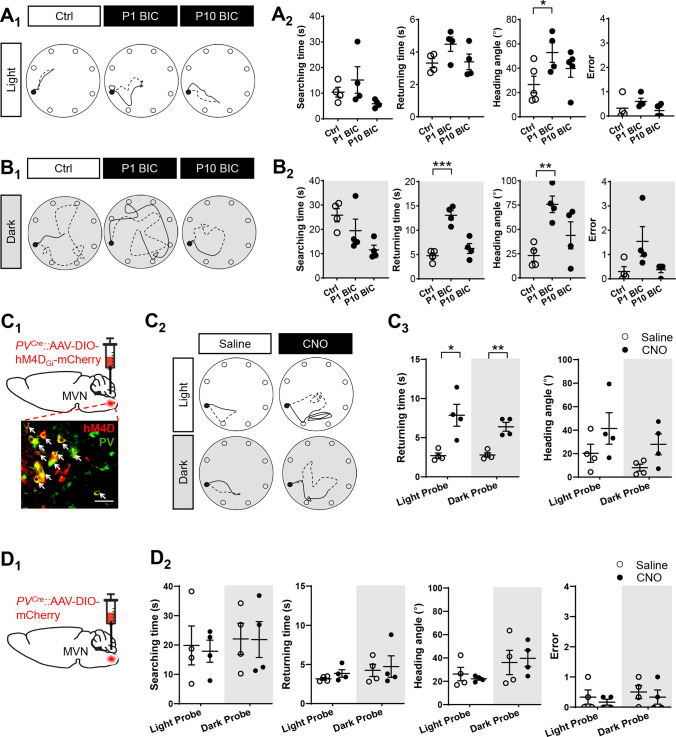
PV-expressing VN neurons are required for navigation in adults. **A**_**1**_, **B**_**1**_ Representative searching (dashed line) and returning (solid line) paths of adult (P60) mice pre-treated with saline as control group (left) or with BIC at P1 (middle) and P10 (right) under light (**A**_**1**_) and dark probes (**B**_**1**_). Filled black circles indicate the location of home base. **A**_**2**_, **B**_**2**_ Graph showing the average searching time (first column), returning time (second column), heading angle (third column), and errors in locating the home base (fourth column) of these mice. *P < 0.05, **P < 0.01, ***P < 0.001 indicate significant differences between the control and BIC-treated groups, one-way ANOVA. **C**_**1**_ Schematic diagram illustrating the injection of AAV5-hSyn-DIO-hM4D_Gi_-mCherry into the MVN of PV-Cre mice at P42. PV-expressing MVN neurons (white arrows) with viral transfection was observed 2 weeks after injection. Scale bar, 20 μm. **C**_**2**_ Trajectories of representative searching (dashed line) and returning (solid line) paths of adult PV-Cre mice expressing hM4D_Gi_ before (left column) and after (right column) CNO administration. Paths in both light (upper panel) and dark (lower panel) probe tests are shown. **C**_**3**_ Chemogenetic inhibition of PV-expressing neurons in the MVN of adult mice increased average returning time, demonstrating the importance of PV-expressing neuron activity in spatial cognitive behavior. *P < 0.05, **P < 0.01, paired t-test. **D**_**1**_ Schematic diagram illustrating the injection of control virus (AAV5-hSyn-DIO-mCherry) into the MVN of PV-Cre mice at P42. **D**_**2**_ No statistical difference (paired t-test) in average searching time (first column), returning time (second column), heading angle (third column), and errors in locating the home base (fourth column) between saline and CNO treatment to these control adult mice. Data are presented as mean ± SEM

To rule out the potential differences in the effect of early postnatal GABA_A_R blockade on circuit maturation between rats and mice, we performed similar navigational tests on P60 mice that were pre-treated with either saline or BIC intervention at P1 or P10. In light probe test (Fig. 4A), BIC treatment at P1 when compared to saline controls resulted in an increased heading angle (P1 BIC: 52.8° ± 8.09° vs. control: 26.57° ± 6.87°; *P* = 0.04), but did not significantly affect their searching time (P1 BIC: 15.17 ± 5.18 s vs. control: 10.40 ± 1.91 s; *P* = 0.42), returning time (P1 BIC: 4.48 ± 0.44 s vs. control: 3.32 ± 0.29 s; *P* = 0.07) or number of errors (P1 BIC: 0.60 ± 0.13 vs. control: 0.32 ± 0.23; *P* = 0.34). In dark probe tests (Fig. 4B), the navigational deficits resulted from P1 BIC pre-treatment became more pronounced, as evidenced by increased returning time (P1 BIC: 13.08 ± 0.93 s vs. control: 4.74 ± 0.59 s; *P* < 0.001) and heading angle (P1 BIC: 75.73° ± 8.56° vs. control: 23.07° ± 5.84°; *P* = 0.002) despite no significant difference in searching time (P1 BIC: 19.42 ± 4.80 s vs. control: 25.83 ± 2.75 s; *P* = 0.29) and number of errors (P1 BIC: 1.54 ± 0.60 vs. control: 0.30 ± 0.20; *P* = 0.10). In contrast, adult mice pre-treated with BIC at P10 did not exhibit navigational impairments in both light and dark probe tests when compared to saline controls, in terms of searching time (light: 5.91 ± 0.75 s; dark: 11.57 ± 1.92 s), returning time (light: 3.39 ± 0.48 s; dark: 6.23 ± 1.03 s), heading angle (39.77° ± 7.27° in light; 43.77° ± 13.92° in dark), and errors (0.23 ± 0.13 s in light; 0.37 ± 0.12 in dark). In all, P60 mice pre-treated with BIC at P1 showed navigational deficits similar to adult rats pre-treated with BIC at P1, suggesting that early postnatal GABA_A_R had key roles in maturation of vestibular circuits in both species.

We reason that PV-expressing neurons, being the most abundant inhibitory interneuron subtype in the adult VN [18], were the most likely contributors to inhibition in the MVN. PV-Cre mice were used to allow silencing of PV-expressing neurons via Cre-mediated expression of chemogenetic inhibitor hM4D_Gi_ in these neurons within the adult MVN (Fig. 4C_1_). Mice displayed similar homeward navigation behavior as rats in dead reckoning test (Fig. 4A) [19]. Returning paths of PV-Cre mice were more tortuous with inhibition of PV-expressing neurons using CNO in both light and dark probe tests (Fig. 4C_2_). In the light probe test, injection of CNO significantly increased the returning time to 7.86 ± 2.79 s (*P* < 0.05, Fig. 4C_3_) when compared to control with saline injection (2.70 ± 0.68 s). In the dark probe test, mice also had difficulty in finding their way back home after inhibition of PV neuron activity in the MVN (6.39 ± 1.12 s with CNO vs. 2.80 ± 0.55 s without CNO, *P* < 0.01, Fig. 4C_3_). The heading angle was unchanged after CNO administration (light probe test: 20.29 ± 7.69 vs. 41.42° ± 13.38°, *P* = 0.220; dark probe test: 8.04 ± 2.96 vs. 27.92° ± 8.93°, *P* = 0.079, Fig. 4C_3_). These demonstrated that PV-expressing interneuron activity in MVN circuitry is required for effective navigation, and that disinhibition of MVN circuits by silencing PV-expressing interneurons led to derangement in navigation behavior.

Notably, navigational behaviour was unchanged in PV-cre mice with control virus after either saline or CNO administration (Fig. 4D_1_). In light test (Fig. 4D_2_), no significant difference was observed in searching time (saline: 19.84 ± 6.33 s vs. CNO: 17.86 ± 3.76 s, *P* = 0.42), returning time (saline: 3.16 ± 0.17 s vs. CNO: 3.85 ± 0.48 s, *P* = 0.22), heading angle (saline: 26.17° ± 5.78° vs. CNO: 22.10° ± 1.17°, *P* = 0.52), or error (saline: 0.33 ± 0.23 vs. CNO: 0.17 ± 0.10, *P* = 0.54). In dark probe tests test (Fig. 4D_2_), navigational performance of mice injected with control virus also did not show difference between saline and CNO administration in searching time (saline: 22.08 ± 5.30 s vs. CNO: 21.85 ± 6.13 s, *P* = 0.98), returning time (saline: 4.25 ± 0.78 s vs. CNO: 4.75 ± 1.37 s, *P* = 0.77), heading angle (saline: 36.09° ± 10.44° vs. CNO: 39.70° ± 7.02°, *P* = 0.78), and error (saline: 0.50 ± 0.22 vs. CNO: 0.33 ± 0.24, *P* = 0.62). These confirmed that CNO did not directly affect navigation performance.

### PV and PNN expression in the MVN of postnatal rats after neonatal treatment with BIC

Does BIC therefore exert its effect on MVN function through altering the development of PV neurons? To test this, immunohistochemistry was conducted to determine whether BIC pre-treatment at P1 reduced the number of PV-expressing neurons.

**Fig. 5:**
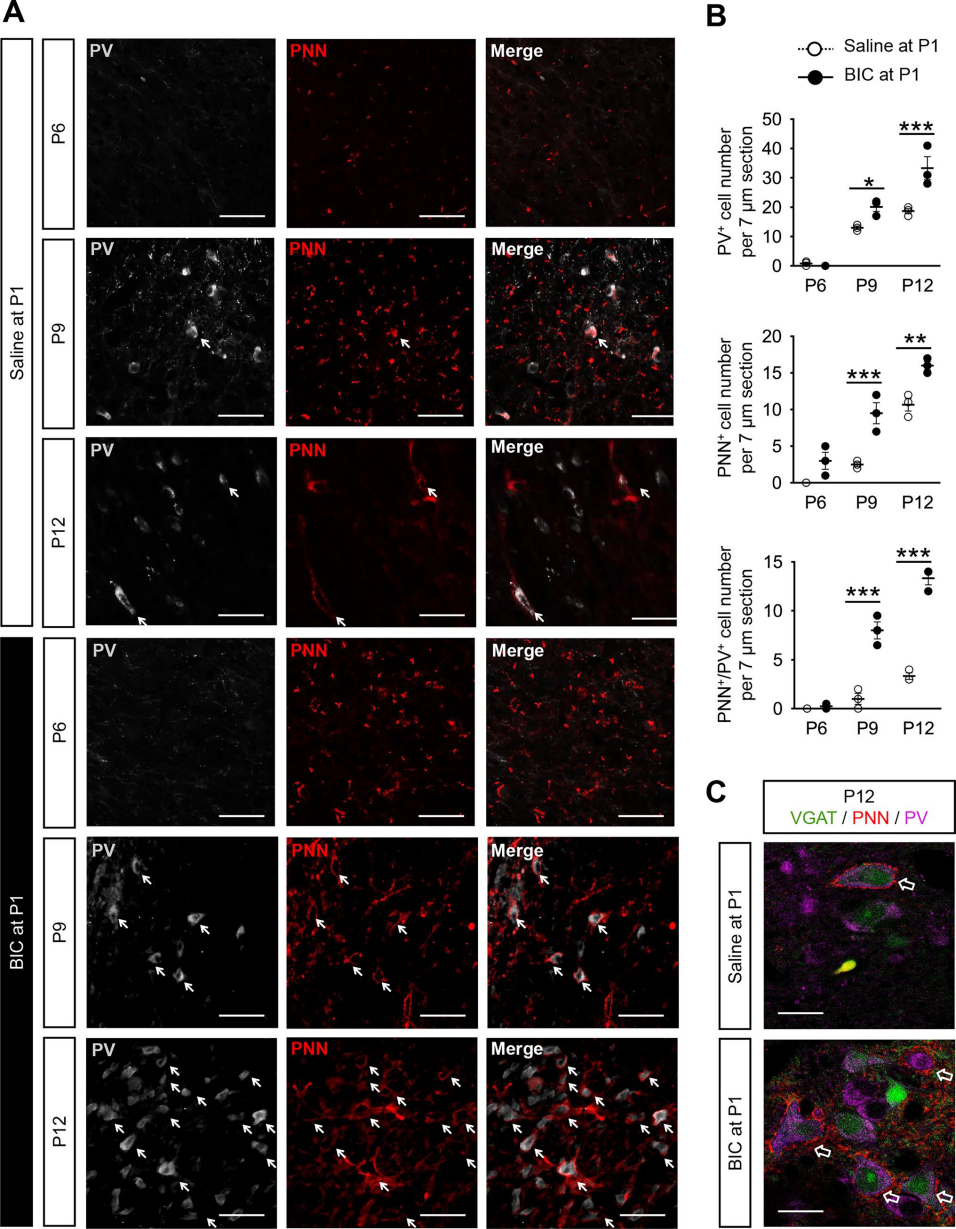
PV-expressing VN neurons increased after BIC treatment at P1. **A** Representative images of PV-expressing (gray), PNN-bearing (red) and PNN-enwrapped PV-expressing cells (solid arrows) in coronal MVN slices (7 μm) of P6 (upper row), P9 (middle row), and P12 (lower row) rats with pre-treatment of saline or BIC at P1. Arrows indicate MVN cells expressing both PV and PNN. **B** The number of PV-expressing cells (upper panel), PNN-enwrapped cells (middle panel), and PNN-enwrapped PV-expressing cells (lower panel) in 7 μm sections of MVN from P9 and P12 rats that were pre-treated with BIC (filled circle) was increased compared to those pre-treated with saline at P1 (open circle). *P < 0.05, **P < 0.01, ***P < 0.001, two-way ANOVA. **C** Representative images showing the expression of PV, PNN and VGAT in the MVN cells of P12 rats pre-treated with saline or BIC at P1. Opened arrows indicate MVN cells co-expressing PV, PNN and VGAT. Data are presented as mean ± SEM. Scale bar: 50 μm (**A**, **C**)

We first characterized the expression pattern of PV-expressing neurons in the MVN of P6, P9 and P12 rats (Fig. 5A), and found gradual increase in number of PV-expressing cells from P6 (0.83 ± 0.54 cells/7 µm section) to P9 (13.00 ± 0.82 cells/7 µm section) and P12 (18.80 ± 0.84 cells/7 µm section) (Fig. 5B). In rats with BIC-treatment at P1, PV-expressing cells were also hardly observed at P6, but were unexpectedly increased significantly in the MVN at both P9 (20.17 ± 1.56 cells/7 µm section; *P* < 0.05) and P12 (33.33 ± 3.97 cells/7 µm section; *P* < 0.05) when compared with controls (Fig. 5C).

As the overall level of inhibition was decreased in rats pre-treated with BIC, were these increased PV-expressing neurons somehow inactive? Consolidation of perineuronal nets (PNN) around PV-expressing neurons is characteristic of high level of neuronal activity in the cortex [27–30]. Using Wisteria floribunda agglutinin (WFA) to label PNNs, we showed that PNNs were not present in MVN neurons of control rats until P9 (2.5 ± 0.5 cells/7 µm section), followed by an increase in the number of PNN-bearing cells in the MVN of P12 rats (10.67 ± 0.88 cells/7 µm section) (Fig. 5C). In rats receiving implantation of BIC-loaded Elvax slice at P1, PNNs in the MVN emerged much earlier at P6 (3.0 ± 1.2 cells/7 µm section), and the number of PNN-enwrapped cells were significantly increased at both P9 (9.5 ± 1.5 cells/7 µm section; *P* < 0.05) and P12 (16.0 ± 0.6 cells/7 µm section; *P* < 0.01) when compared with the control group (Fig. 5C).

More specifically, no PV-expressing neurons enwrapped by PNN were observed in control rats at P6, only 1 ± 1 neuron/7 µm section at P9 and 3.33 ± 0.33 neurons/7 µm section at P12 (Fig. 5A). In rats with pre-treatment of BIC at P1, more PV-expressing neurons were surrounded by PNNs in the MVN at P9 (8 ± 1 cells/7 µm section; *P* < 0.05) and P12 (13.33 ± 0.67 cells/7 µm section; *P* < 0.001) when compared with the controls (Fig. 5B). Given that consolidation of PNN around PV-expressing neurons is activity-dependent [28] and that PNN-enwrapped PV-expressing neurons in the MVN co-expressed vesicular GABA transporter (VGAT) (Fig. 5C), immunohistochemical results suggested that inhibitory transmission would be enhanced with neonatal BIC treatment. This was at odds with both electrophysiological data observed, and chemogenetic data which showed that spatial cognitive deficits occurred when inhibitory transmission mediated by PV-expressing neurons was reduced. We therefore reasoned that the most probable scenario was that the connectivity of PV-expressing neurons in the MVN was altered by BIC pre-treatment, resulting in null recruitment of these neurons into MVN circuits responsible for spatial navigation.

### Neonatal BIC treatment disrupted PV-expressing interneuron-mediated gating of MVN outputs

To address this, we directly assessed the effect of neonatal BIC treatment on the total amount of inhibition at MVN output neurons that belong to the ascending pathway for spatial cognition. The specific contribution of PV-expressing neurons in providing such inhibition was then revealed by optogenetic-coupled whole-cell patch-clamp recording.

**Fig. 6:**
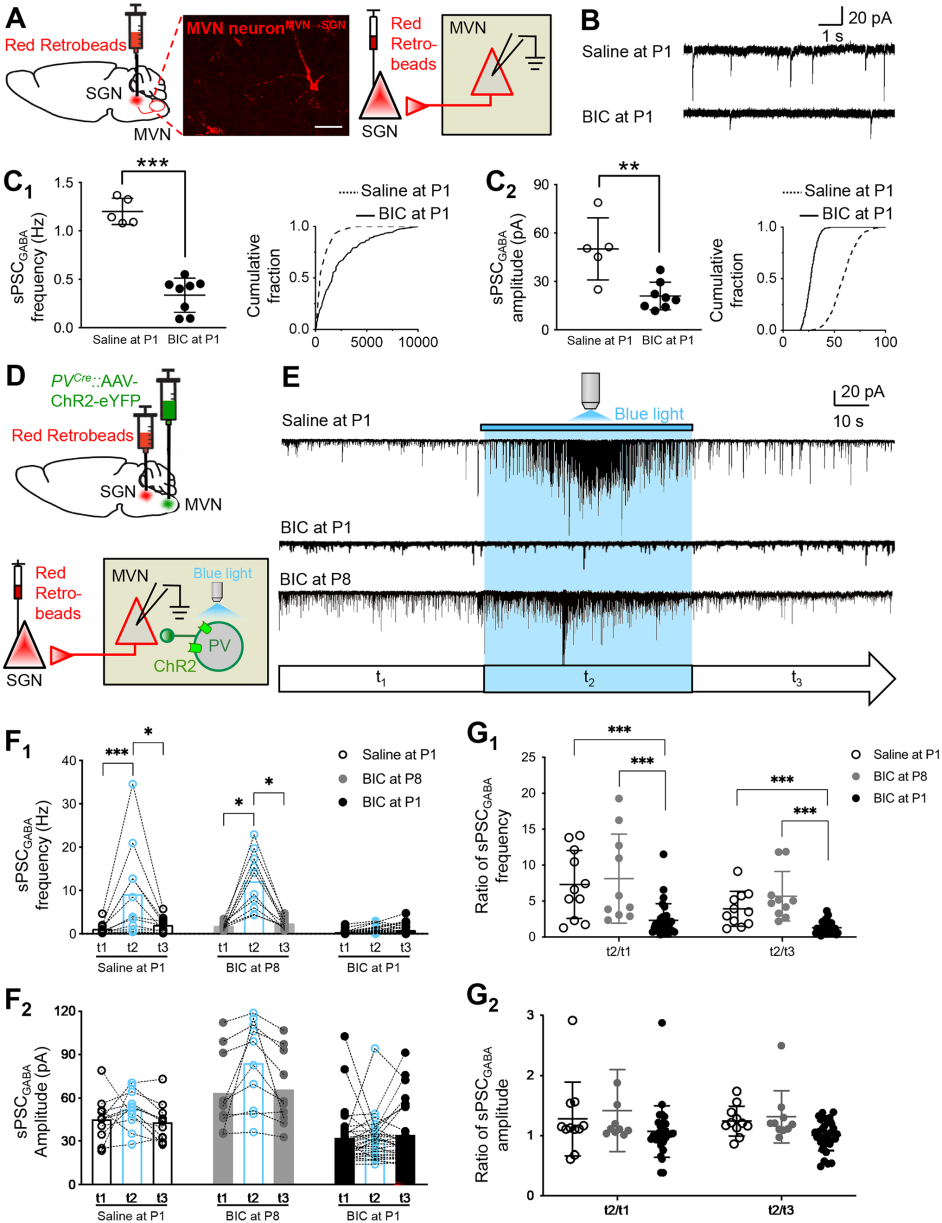
BIC treatment at P1 disrupts hardwiring of inhibitory connections from PV-expressing neurons to MVN outputs. **A** Schematic diagram illustrating the injection of red retrobeads into the SGN 3 days before the recording of SGN-projecting MVN neurons of P60 rats. Representative image shown in the middle panel. Scale bar: 50 μm. **B** Representative traces of sPSC_GABA_ of labelled neurons from P60 rats pre-treated with saline or BIC at P1. **C** Scattered dots showing the frequency (**C**_**1**_) or amplitude (**C**_**2**_) of sPSC_GABA_ in individual neurons from P60 rats pre-treated with saline (open circle) or BIC (filled circle) at P1. Cumulative fractions of IEI or amplitude of recorded sPSC_GABA_ for each treatment are shown. **P < 0.01, ***P < 0.001, t-test. **D** Schematic diagram showing the recording of sPSC_GABA_ from SGN-projecting MVN neurons of P60 PV-cre mice, with optogenetic activation of ChR2-expressing PV interneurons. **E** Representative tracings of sPSC_GABA_ recorded from SGN-projecting MVN neurons before (t_1_), during (t_2_, light blue), and after (t_3_) blue light stimulation in P60 mice pre-treated with saline or BIC at P1. **F**_**1**_ Frequency of sPSC_GABA_ recorded from P60 mice pre-treated with BIC (filled column) at P1 was significantly decreased during optogenetic stimulation, compared to P60 mice pre-treated with saline (open column) at P1. **F**_**2**_ Amplitude of sPSC_GABA_ remained unchanged. **G** t_2_/t_1_ and t_2_/t_3_ ratios of sPSC_GABA_ frequency (**G**_**1**_) or amplitude (**G**_**2**_) indicate decreased response to opto-activation of PV-expressing neurons. *P < 0.05, ***P < 0.001, one-way ANOVA. Data are presented as mean ± SEM

MVN neurons projecting to SGN were identified by retrograde labelling from the SGN (Fig. 6A, Supplementary Fig. 2A). This allowed specific recording of sPSC_GABA_ in these MVN output neurons at P60. We found that both the frequency (1.2 ± 0.06 vs. 0.34 ± 0.06 Hz; *P* < 0.001) and the amplitude (50.17 ± 8.61 vs. 20.98 ± 3.03 pA; *P* < 0.01) of sPSC_GABA_ were significantly decreased in SGN-projecting MVN neurons of P60 rats with pre-treatment of BIC at P1 when compared with controls (Fig. 6B, C_1,2_). The IEI curve of SGN-projecting MVN neurons of BIC-treated rats was shifted to the right relative to that of controls, while the fraction curve of sPSC_GABA_ amplitude was shifted to the left (Fig. 6C_1,2_). These results were in line with developmental decrease in sIPSCs frequency and amplitude in BIC-treated rats (Fig. 2B_2,3_, Supplementary Fig. 1B), implying that neonatal GABA interference shifted the developmental trajectory of MVN circuitry to a more excited state.

To show the specific contribution of PV-expressing interneurons to reduction of PSC_GABA_ at SGN-projecting MVN neurons, optogenetic-coupled whole-cell patch-clamp recording was conducted in 3 groups of PV-Cre mice, viz. pre-treated with saline at P1 (control), BIC at P1, and BIC at P8 (Fig. 6D). sPSC_GABA_ at these projecting neurons were recorded at P60 when PV-expressing interneurons which expressed ChR2 were activated by blue light (Fig. 6E). As expected, the frequency of sPSC_GABA_ at SG-projecting MVN neurons of control mice was dramatically increased upon optogenetic activation of PV-expressing MVN neurons with blue light, and returned to the pre-stimulation level when the blue light was switched off (t_1_: 1.15 ± 0.39 vs. t_2_: 9.14 ± 3.22 vs. t_3_: 2.04 ± 0.53 Hz, *P* < 0.001; t_1_ vs t_2_: *P* < 0.001, t_1_ vs t_3_: *P* = 0.41, t_2_ vs t_3_: *P* < 0.05, Fig. 6F_1_). In adult mice pre-treated with BIC at P1, however, blue light stimulation of PV-expressing neurons in the MVN did not change the frequency of sPSC_GABA_ of SGN-projecting neurons (t_1_: 0.39 ± 0.08 vs. t_2_: 0.72 ± 0.14 vs. t_3_: 0.89 ± 0.20 Hz, *P* = 0.45, Fig. 6F_1_). Notably the frequency (t_1_: 1.82 ± 0.25 vs. t_2_: 12.12 ± 2.11 vs. t_3_: 2.41 ± 0.40 Hz, *P* = 0.46, Fig. 6F_1_) of sPSC_GABA_ in mice pre-treated with BIC at P8 were not different when compared to controls. On the other hand, the amplitude of sPSC_GABA_ remained unchanged before, during and after stimulation with blue light in all groups (control group: 45.18 ± 4.60 vs. 52.22 ± 3.97 vs 43.04 ± 4.00 pA, *P* = 0.15; BIC-treated at P1: 32.11 ± 3.26 vs. 30.4 ± 2.83 vs. 33.74 ± 3.55 pA, *P* = 0.66; BIC-treated at P8: 63.67 ± 8.61 vs. 83.81 ± 9.66 vs. 65.83 ± 8.12 pA, *P* = 0.15, Fig. 6F_2_). This indicated that BIC exposure after P8 did not change the connectivity of PV-expressing neurons onto MVN outputs.

**Fig. 7:**
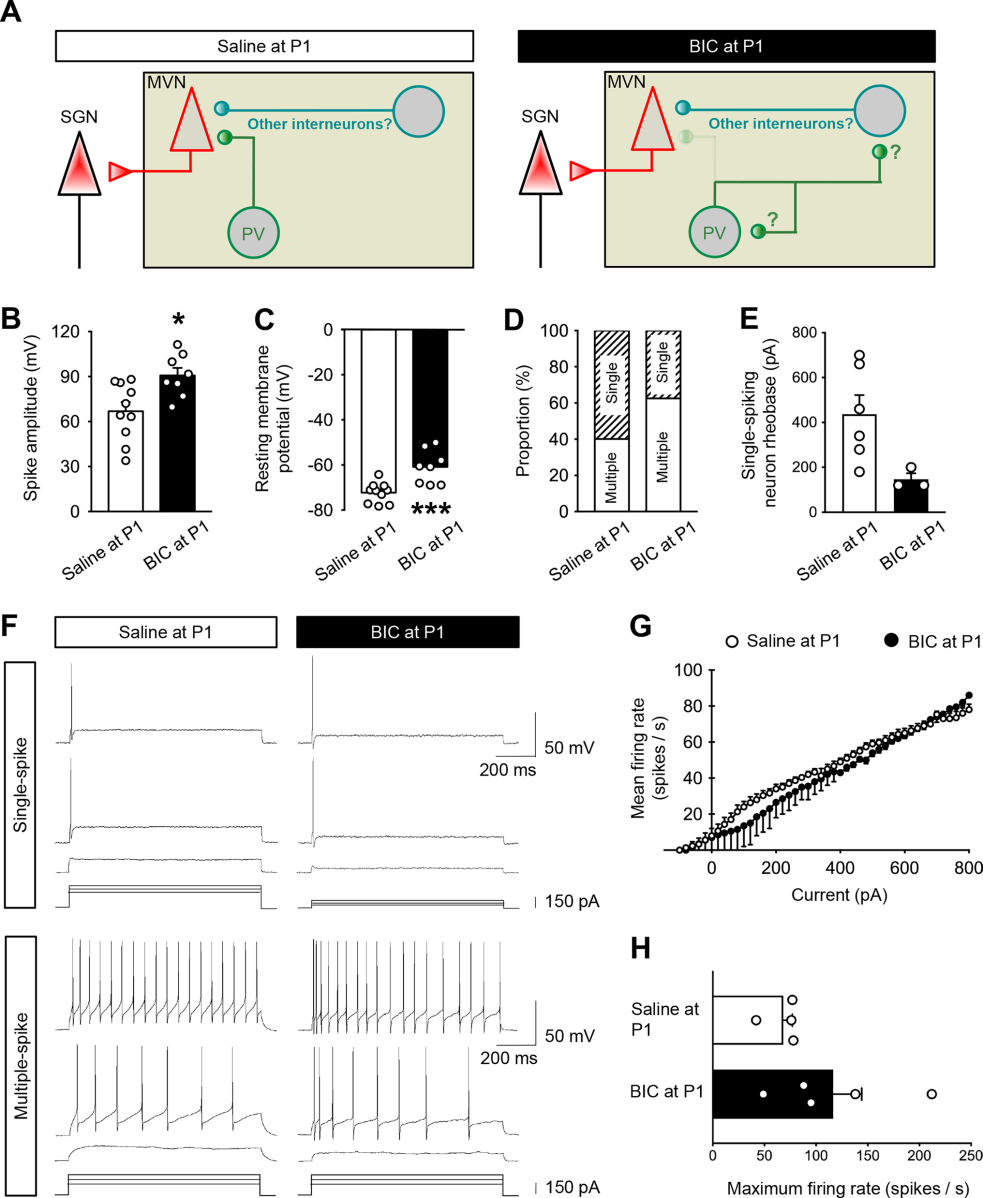
Altered gating resultant from suppression of neonatal GABAergic transmission distorts VN output characteristics. **A** Schematic diagram illustrating shift of connectivity of PV-expressing interneurons from projecting neurons (left) to other interneurons in the MVN (right) after BIC pre-treatment at P1. **B** Averaged amplitude of action potentials from adult MVN neurons after neonatal saline or BIC treatment. **C** Resting membrane potential of MVN neurons. **D** Percentage of single-spiking (shaded column) vs. multiple-spiking (open column) MVN neurons in control or BIC-treated rats. **E** Rheobase of single-spiking neurons from control or BIC-treated rats. **F** Examples of membrane responses of single-spike (upper panel) or multiple-spike (bottom panel) SGN-projecting MVN neurons from P60 rats pre-treated with saline (left panel) or BIC (right panel) at P1. Responses were elicited by injection of three current steps of increasing amplitude. **G** Gains of input–output curve from multiple-spike firing SGN-projecting MVN neurons between P60 rats pre-treated at P1 with saline (open circle) and those with BIC (filled circle). **H** Maximum firing rate of SGN-projecting MVN neurons from P60 rats pretreated with saline or BIC at P1. *P < 0.05, t-test. Data are presented as mean ± SEM

To better illustrate the effect of optogenetic activation of PV-expressing interneuron on sPSC_GABA_ in individual SGN-projecting neurons, we calculated the ratio of t_2_/t_1_ (the ratio of the frequency or amplitude of sPSC_GABA_ during blue light stimulation to that before stimulation) and the ratio of t_2_/t_3_ (the ratio of the frequency or amplitude of sPSC_GABA_ during blue light stimulation to that when the stimulation was removed) of individual neurons. Both t_2_/t_1_ and t_2_/t_3_ of sPSC_GABA_ frequency were significantly decreased in SGN-projecting MVN neurons of adult mice with pre-treatment of BIC at P1 when compared with controls (t_2_/t_1_: 7.31 ± 1.43 vs. 2.26 ± 0.41, *P* < 0.001; t_2_/t_3_: 3.92 ± 0.73 vs. 1.25 ± 0.16, *P* < 0.001, Fig. 6G_1_), but not in mice pre-treated with BIC at P8 (t_2_/t_1_: 8.13 ± 1.96, *P* = 0.41; t_2_/t_3_: 5.68 ± 1.09, *P* = 0.12, Fig. 6G_1_). No significant change was found in t_2_/t_1_ or t_2_/t_3_ of sPSC_GABA_ amplitude of SGN-projecting neurons in P60 mice pre-treated with BIC at P1 or P8 when compared with those of control mice (t_2_/t_1_: 1.28 ± 0.19 vs. 1.42 ± 0.22 vs. 1.03 ± 0.08, *P* = 0.18; t_2_/t_3_: 1.24 ± 0.08 vs. 1.32 ± 0.14 vs. 0.97 ± 0.06; *P* = 0.12, Fig. 6G_2_). Results revealed a critical time window before P8 when GABAergic transmission is necessary for consolidation of PV-expressing interneuron-mediated gating of MVN output (Fig. 7A).

### Firing properties of SGN-projecting MVN neurons of adult rats pre-treated with BIC at P1

Given reduction of inhibition mediated by PV-expressing interneurons at SGN-projecting neurons within the adult MVN after neonatal BIC treatment, we asked whether firing properties of these projecting neurons were changed. The averaged amplitude of action potentials from MVN neurons in P60 rats pre-treated with BIC at P1 (90.77 ± 4.98 mV) were significantly higher than those from control rats (66.86 ± 6.06 mV; *P* < 0.05) (Fig. 7B). Also, the resting membrane potential of MVN neurons from P60 rats pre-treated with BIC (− 60.84 ± 2.65 mV) was higher than that from controls (− 72.28 ± 1.41 mV; *P* < 0.001) (Fig. 7C). On the basis of responses to current steps, we further classified cells as either single-spiking or multiple-spiking MVN neurons. Sixty percent of SGN-projecting neurons in the MVN were single-spiking neurons in the controls (n = 10 cells), whereas the majority of recorded cells (62.5%) were multiple-spiking neurons in the MVN of BIC-treated rats (n = 8 cells) (Fig. 7D). For single-spiking neurons, those from the BIC-treated rats had a lower rheobase (146.67 ± 26.67 pA) when compared with control rats (436.67 ± 85.54 pA; *P* < 0.05), but possessed a comparable amplitude in the first action potential (64.86 ± 10.4 vs. 81.16 ± 1.66 mV; *P* = 0.19) (Fig. 7E and Supplementary Table 2). For multiple-spiking MVN neurons, they increased the firing rate in response to increasing depolarizing current steps (Fig. 7F). These neurons from BIC-treated rats had identical gains of input–output (I-O) curve (0.098 ± 0.012 vs. 0.0925 ± 0.01 spikes/s/pA; *P* = 0.71) (Fig. 7G), and comparable maximum firing rate with controls (68.25 ± 8.76 vs. 116.4 ± 27.76 spikes/s; *P* = 0.11) (Fig. 7H).

These results therefore show that the excitability and firing of SGN-projecting MVN neurons were increased after neonatal BIC-treatment due to long-lasting decrease in inhibitory gating by PV-expressing neurons. This revealed that early GABAergic transmission is necessary for formation of inhibitory connections that shape VN output along ascending pathways for spatial navigation.

## Discussion

We demonstrated the requirement of neonatal GABA transmission for development and maturation of VN circuitry in support of adult navigational behaviour. Specifically, activity of PV-expressing neurons in VN circuits was crucial for navigation in adulthood. Neonatal interference of GABAergic transmission in the MVN decreased connectivity between PV-expressing interneurons and SGN-projecting neurons in the adult MVN. This led to reduced inhibition and consequently increased excitability of these projecting neurons. Results demonstrated that perturbed GABAergic transmission deranged assembly of inhibitory PV-expressing VN interneuron circuits and distorted VN output to higher centers that support spatial navigational behaviour.

Spatial cognition depends on neural representations of space built from sensory and self-motion cues [31]. Without sensory cues from the environment, such as when animals are placed in the dark, self-motion cues generated from vestibular input become dominant. Linear and angular acceleration inputs, derived respectively from the otolith organs and semicircular canals [31], are processed by MVN circuits before transmission via the SGN to higher brain regions for spatial cognition [10, 32–37]. Impairment in spatial navigation performance of adult rats pre-treated with BIC at P1 could be compensated by augmentation with visual cues in the light probe test when the rodent was in motion. These demonstrated that local BIC treatment in the neonatal MVN achieved long-lasting disruption of the vestibular circuits that support navigational performance. Similar pre-treatment of VN with BIC at P8 or P14 did not affect performance in either light or dark probe test. This suggested that spatial reckoning circuits no longer required vestibular input after P8 for subsequent maturation and thus were not affected by transient BIC-induced perturbations in VN function. Our results therefore revealed a critical period ending before P8, during which reduced GABAergic transmission in MVN significantly impacts adult acquisition of spatial navigation.

Poor performance in the new location test by adult rats pre-treated with BIC at P1 provided evidence for dysfunction in the establishment of spatial memory [38]. Such deficit was also present in adult rats pre-treated with BIC at P8, despite normal performance in light and dark probe tests. This suggested that higher centres, such as the hippocampal place cells that store spatial memories [39], were still susceptible to distorted inputs from MVN beyond P8. Deficits in new location test but not other navigational tasks further suggested that neurons belonging to the posterior vestibulo-thalamocortical pathway which support spatial memory in the hippocampus [32], become consolidated at a later developmental stage than those in the anterior pathway that generate head direction signals used for navigation [32]. For adult rats receiving BIC treatment at P14, no derangement was observed in any of the dead reckoning tests. This implied that all ascending connections for spatial cognition were mature by then. Inputs from MVN were therefore no longer required for consolidation of these circuits. The multiple time points at which different levels of spatial cognitive function became mature, as revealed by the aforementioned tests, mirror the sequential maturation of the head direction cell, place cell and grid cell systems [12, 40] for the hierarchical representation of space.

Persistent decrease of inhibitory transmission in the MVN of rats pre-treated with BIC at P1 showed that neonatal blockade of GABAergic transmission stunted maturation of GABAergic circuits within the MVN. Notably, accelerated maturation of GABAergic transmission, such as by premature conversion of early depolarizing GABA to hyperpolarizing with bumetanide, delayed development of the excitatory system but did not affect the inhibitory system [41]. This further highlights the unique role of early depolarizing GABAergic transmission in directing formation of brain circuits.

Synaptic plasticity underlies motor learning in the VN [42]. Suppressed inhibitory transmission kickstarts bouts of critical period-like synaptic plasticity in the visual cortex [43, 44]. Similarly, when GABAergic transmission in VN was dampened by pre-treatment with BIC at P1, LTD_GABA_ was observed in almost twice the number of VN neurons at P9 and P14 compared to age-matched controls (Fig. 3D). This revealed an extended duration of critical period plasticity beyond P14 in rats pre-treated with BIC at P1, contrasting closure of critical period by P14 in control rats. It was reported that BIC not only acts against GABA_A_R but also blocks small conductance calcium-activated potassium channels (SK) channels, potentially leading to a reduction in afterhyperpolarization (AHP) and facilitation of LTD [45]. However, immunofluorescence staining of MVN tissue sections from P1 rodents revealed abundant expression of GABA_A_R but only spare SK channels (Supplementary Fig. 2). Coupled with the fact that the characteristics of AHP from SGN-projecting MVN neurons remain unchanged in BIC-treated versus control rats (Supplementary Table 2), our findings support that the long-lasting effects of neonatal BIC intervention on neural circuit formation are primarily resulted from perturbations to the GABAergic system. We therefore reason that extraneous plasticity from sustained depression of GABAergic transmission during the critical period deranges synaptic connectivity and delays maturation in the VN, as in cortical circuits [46, 47].

While inhibitory circuits in MVN are known to be required for vestibular function [48–50], the specific interneuron subtype responsible was previously unknown. Our chemogenetic experiments revealed the PV-expressing inhibitory interneurons to be key for navigational behaviour. Unexpectedly, pre-treatment with BIC at P1 resulted in a significant increase in both the number of PV-expressing inhibitory interneurons and the percentage of PV-expressing neurons enwrapped by PNN (Fig. 5). Given that PNN formation is activity-dependent [28], increased consolidation of PNN suggested that these PV-expressing interneurons were active, which may be the result of increased excitatory drive (E/I ratio) within VN circuits after neonatal perturbation of GABAergic transmission.

The only way by which increased population of active inhibitory PV-expressing interneurons (Fig. 5) could lead to reduced inhibition of MVN outputs (Fig. 6E) was if they were not connected to the output neurons of the MVN. To test this, we probed the connectivity of PV-expressing neurons in the VN by optogenetics-coupled whole-cell patch-clamp recording. Opto-activated sPSC_GABA_ was no longer recorded at SGN-projecting MVN neurons in adult mice pre-treated with BIC at P1. This proved that PV-expressing interneurons no longer directly inhibit these projecting neurons after BIC-treatment (Fig. 7A), resulting in disinhibition of the SGN-projecting neurons in the ascending pathway for spatial navigation (Fig. 6E). Notably, while all PSC_GABA_ resulting from PV-expressing neurons was eliminated at MVN output neurons by BIC-pretreatment of rats at P1 (Fig. 7A), the total amount of sPSC_GABA_ was only decreased by 70% (Fig. 6F). This suggested that while the majority of inhibitory gating of MVN output to SGN was contributed by PV-expressing interneurons in normal rats (Fig. 7A), other yet to be identified inhibitory interneuron subtypes also contributed to feedforward gating of VN outputs. Nonetheless, these residue inhibitory inputs were insufficient to support efficient vestibular-dependent spatial navigational behaviour.

Increased intrinsic excitability and firing of the SGN-projecting MVN neurons was consistent with loss of inhibitory gating by PV-expressing neurons after neonatal exposure to BIC. The resultant spiking pattern in the MVN of rodents pre-treated with BIC was predominated by multiple spikes. This deviated from single spikes predominantly observed in sensory circuits [51, 52], reflecting distorted spiking dynamics in MVN outputs along the ascending pathway for vestibular-dependent spatial cognition [53–55]. Given the limited sample size and the lack of subgroup analysis based on cell types, it is worthwhile for future studies to explore the specific impact of neonatal intervention of GABAergic activity on the electrophysiological properties of distinct cell types.

Our results reveal the critical role of GABAergic transmission before P8 in allowing entrainment of developing MVN circuits via early vestibular inputs. Whether the observed navigational deficits in adults result from distorted MVN output and/or from incomplete integration of vestibular signals with spatial cognitive circuits in higher centers remains to be elucidated. Nonetheless, failure of other sensory input to compensate for deranged vestibular signals emphasizes the critical role of PV-expressing interneurons in shaping MVN output to support spatial navigation.

### Supplementary Information

Below is the link to the electronic supplementary material.

Supplementary file1 (DOCX 22 KB)

Supplementary file2 (DOCX 17 KB)

Supplementary file3 (DOCX 26 KB)

Supplementary file4Supplementary Fig. 1. Fold change in sPSC profiles after BIC treatment at P1. (A) Ratio of the average frequency of sPSCs (triangle for sEPSCs; inverted triangle for sIPSCs). Data from P1 control rats (i.e. without surgery) were set as the base. Data from P8 and P14 rats were calculated by comparing BIC and saline treatment of the respective age group (the corresponding data shown in Fig. 2A2, 2B2). (B) Ratio of the average amplitude of sPSCs (triangle for sEPSCs; inverted triangle for sIPSCs). Data from P1 control rats (i.e. without surgery) were set as the base. Data from P8 and P14 rats were calculated by comparing BIC and saline treatment of the respective age group (the corresponding data shown in Fig. 2A3, 2B3) (TIF 1166 KB)

Supplementary file5Supplementary Fig. 2. Immunohistological and electrophysiological validation of effective retrograde tracing cum optogenetic-coupled whole-cell patch-clamp recording. (A1) Representative image showing the injection site of retrograde tracer (CTB) deposited into the SGN 3 days before recording. Abbreviation: IV, fourth ventricle. Scale bar: 200 μm. (A2) Representative images of MVN demonstrating successful retrograde labeling of CTB and its colocalization with glutamatergic neuronal marker VGluT1. Scale bar: 50 μm. (B) Injection site of AAV5-DIO-ChR2-eYFP in MVN of PV-Cre mice (upper panel; scale bar: 50 μm). PV neurons with viral transfection, viewed under fluorescent (lower left panel) and bright-field microscopy (lower middle panel; scale bar: 10 μm). Recording pipette was shown in lower middle panel. (C) Voltage response recorded from ChR2-expressing neurons upon 1 and 2 Hz optical stimulation (blue bars). (D) Representative images showing immunohistological staining of GABAAR and SK2 channels (KCNN2) in MVN of a P1 control mouse. Scale bar: 20 μm (TIF 7631 KB)

## Data Availability

The data that support the findings of this study are available from the corresponding authors upon reasonable request.
