# Supplementary material for: Neonatal GABAergic transmission primes vestibular gating of output for adult spatial navigation
Source: Cell Mol Life Sci. 2024 Mar 19;81(1):147. doi: 10.1007/s00018-024-05170-x (PMC10951018; doi:10.1007/s00018-024-05170-x)
Supplement: Supplementary file 1 — Supplementary file1 (DOCX 22 KB) [file 18_2024_5170_MOESM1_ESM.docx]

**Supplementary Table 1:** Number of rodents used.

|  | | Elvax implant at P1 | | Elvax implant  at P8 | | Elvax implant  at P10 | | Elvax implant  at P14 | | Virus injection  at P42 | | Control | |
| --- | --- | --- | --- | --- | --- | --- | --- | --- | --- | --- | --- | --- | --- |
|  |  | Saline | BIC | Saline | BIC | Saline | BIC | Saline | BIC | Saline | CNO |  |  |
| Navigational test at P60 | Elvax implantation (SD Rats) | 8 | 5 | 4 | 5 |  |  | 4 | 6 |  |  |  |  |
|  | Elvax implantation (C57BL/6J Mice) | 4 | 4 |  |  | 4 | 4 |  |  |  |  | 4 | 4 |
|  | Chemogenetic  perturbation (PV-Cre Mice) |  |  |  |  |  |  |  |  | 4 | 4 |  |  |
| Immunohistochemical staining | (SD Rats) | P6 P9 P12 | P6 P9 P12 |  |  |  |  |  |  |  |  |  |  |
|  |  | 2 3 3 | 3 3 3 |  |  |  |  |  |  |  |  |  |  |
|  | (C57BL/6J Mice) |  |  |  |  |  |  |  |  |  |  | P1 | P60 |
|  |  |  |  |  |  |  |  |  |  |  |  | 2 | 3 |
| Electrophysiology | LTDGABA (SD Rats) | P5 P9 P14 | P5 P9 914 |  |  |  |  |  |  |  |  |  |  |
|  |  | 5 6 6 | 3 3 4 |  |  |  |  |  |  |  |  |  |  |
|  | sEPSC/sIPSC (SD Rats) | P1 P8 P14 | P1 P8 P14 |  |  |  |  |  |  |  |  |  |  |
|  |  | 3 3 3 | 3 4 4 |  |  |  |  |  |  |  |  |  |  |
|  | sPSCGABA (SD Rats) | P60 | P60 |  |  |  |  |  |  |  |  |  |  |
|  |  | 4 | 7 |  |  |  |  |  |  |  |  |  |  |
|  | Optogenetic  response (PV-Cre Mice) | P60 | P60 | P60 | P60 |  |  |  |  |  |  | P60 |  |
|  |  | 3 | 3 | 6 | 6 |  |  |  |  |  |  | 2 |  |
