# Supplementary material for: Neonatal GABAergic transmission primes vestibular gating of output for adult spatial navigation
Source: Cell Mol Life Sci. 2024 Mar 19;81(1):147. doi: 10.1007/s00018-024-05170-x (PMC10951018; doi:10.1007/s00018-024-05170-x)
Supplement: Supplementary file 2 — Supplementary file2 (DOCX 17 KB) [file 18_2024_5170_MOESM2_ESM.docx]

**Supplementary Table 2:** Intrinsic properties and firing activity of SGN-projecting neurons in the MVN of P60 rats

| Saline at P1 | Saline at P1 | BIC at P1 | *P* values |
| --- | --- | --- | --- |
| Capacitance (pF) | 80.49±13.72 | 65.81±15.53 | *P* = 0.57 |
| Input resistance (MΩ) | 72.92±13.16 | 78.84±9.49 | *P* = 0.73 |
| Single-spike amplitude (mV) | 64.86±10.4 | 81.16±1.66 | *P* = 0.8 |
| Action potential half-width (ms) | 1.13±0.11 | 1.16±0.11 | *P* = 0.84 |
| Rheobase (pA) | 310±74.37 | 164±33.7 | *P* = 0.21 |
| I-O gain (spike / pA) | 0.1±0.01 | 0.09±0.01 | *P* = 0.7 |
| I-O linear coefficient (R2) | 0.93±0.02 | 0.96±0.01 | *P* = 0.48 |
| Maximum rise slope (mV / ms) | 128.61±22.89 | 137.07±19.6 | *P* = 0.84 |
| Afterhyperpolarization (mV) | 13.12±3.61 | 15.85±3.80 | *P* = 0.70 |

Data are presented as mean±SEM (saline: n = 10 cells; BIC: n = 8 cell; 3 rats in each group).
