# Supplementary material for: Neonatal GABAergic transmission primes vestibular gating of output for adult spatial navigation
Source: Cell Mol Life Sci. 2024 Mar 19;81(1):147. doi: 10.1007/s00018-024-05170-x (PMC10951018; doi:10.1007/s00018-024-05170-x)
Supplement: Supplementary file 3 — Supplementary file3 (DOCX 26 KB) [file 18_2024_5170_MOESM3_ESM.docx]

**Supplementary Table 3: Statistical tests (two-way ANOVA) for Fig. 2A_2-3_, 2B_2-3_, 3F, and 4B.**

| *P* value for **Fig. 2A_2_** | | P1 | P8 | | P14 | |
| --- | --- | --- | --- | --- | --- | --- |
|  |  | Ctrl | Saline at P1 | BIC at P1 | Saline at P1 | BIC at P1 |
| P1 | Ctrl |  | > 0.999 | 0.058 | 0.870 | < 0.001 |
| P8 | Saline at P1 |  |  | 0.049 | 0.945 | < 0.001 |
|  | BIC at P1 |  |  |  | 0.946 | 0.006 |
| P14 | Saline at P1 |  |  |  |  | < 0.001 |
|  | BIC at P1 |  |  |  |  |  |

| *P* value for **Fig. 2A_3_** | | P1 | P8 | | P14 | |
| --- | --- | --- | --- | --- | --- | --- |
|  |  | Ctrl | Saline at P1 | BIC at P1 | Saline at P1 | BIC at P1 |
| P1 | Ctrl |  | > 0.999 | > 0.999 | 0.986 | 0.902 |
| P8 | Saline at P1 |  |  | > 0.999 | 0.999 | 0.989 |
|  | BIC at P1 |  |  |  | 0.999 | 0.994 |
| P14 | Saline at P1 |  |  |  |  | > 0.999 |
|  | BIC at P1 |  |  |  |  |  |

| *P* value for **Fig. 2B_2_** | | P1 | P8 | | P14 | |
| --- | --- | --- | --- | --- | --- | --- |
|  |  | Ctrl | Saline at P1 | BIC at P1 | Saline at P1 | BIC at P1 |
| P1 | Ctrl |  | 0.071 | > 0.999 | 0.281 | 0.999 |
| P8 | Saline at P1 |  |  | 0.041 | > 0.999 | 0.048 |
|  | BIC at P1 |  |  |  | 0.200 | > 0.999 |
| P14 | Saline at P1 |  |  |  |  | 0.050 |
|  | BIC at P1 |  |  |  |  |  |

| *P* value for **Fig. 2B_3_** | | P1 | P8 | | P14 | |
| --- | --- | --- | --- | --- | --- | --- |
|  |  | Ctrl | Saline at P1 | BIC at P1 | Saline at P1 | BIC at P1 |
| P1 | Ctrl |  | 0.991 | > 0.999 | 0.591 | 0.843 |
| P8 | Saline at P1 |  |  | 0.748 | 0.999 | 0.161 |
|  | BIC at P1 |  |  |  | 0.215 | 0.999 |
| P14 | Saline at P1 |  |  |  |  | 0.003 |
|  | BIC at P1 |  |  |  |  |  |

| *P* value for **Fig. 3F** | | P5 | | P9 | | P14 | |
| --- | --- | --- | --- | --- | --- | --- | --- |
|  |  | Saline at P1 | BIC at P1 | Saline at P1 | BIC at P1 | Saline at P1 | BIC at P1 |
| P5 | Saline at P1 |  | < 0.001 | < 0.001 | < 0.001 | < 0.001 | < 0.001 |
|  | BIC at P1 |  |  | < 0.001 | < 0.001 | < 0.001 | < 0.001 |
| P9 | Saline at P1 |  |  |  | < 0.001 | < 0.001 | < 0.001 |
|  | BIC at P1 |  |  |  |  | < 0.001 | < 0.001 |
| P14 | Saline at P1 |  |  |  |  |  | < 0.001 |
|  | BIC at P1 |  |  |  |  |  |  |

| *P* value for **Fig. 4B *top*** | | P6 | | P9 | | P12 | |
| --- | --- | --- | --- | --- | --- | --- | --- |
|  |  | Saline at P1 | BIC at P1 | Saline at P1 | BIC at P1 | Saline at P1 | BIC at P1 |
| P6 | Saline at P1 |  | > 0.999 | 0.007 | < 0.001 | < 0.001 | < 0.001 |
|  | BIC at P1 |  |  | 0.004 | < 0.001 | < 0.001 | < 0.001 |
| P9 | Saline at P1 |  |  |  | 0.021 | 0.500 | < 0.001 |
|  | BIC at P1 |  |  |  |  | > 0.999 | 0.003 |
| P12 | Saline at P1 |  |  |  |  |  | 0.001 |
|  | BIC at P1 |  |  |  |  |  |  |

| *P* value for **Fig. 4B *mid*** | | P6 | | P9 | | P12 | |
| --- | --- | --- | --- | --- | --- | --- | --- |
|  |  | Saline at P1 | BIC at P1 | Saline at P1 | BIC at P1 | Saline at P1 | BIC at P1 |
| P6 | Saline at P1 |  | 0.389 | 0.645 | < 0.001 | < 0.001 | < 0.001 |
|  | BIC at P1 |  |  | > 0.999 | 0.003 | < 0.001 | < 0.001 |
| P9 | Saline at P1 |  |  |  | 0.001 | < 0.001 | < 0.001 |
|  | BIC at P1 |  |  |  |  | 0.999 | 0.003 |
| P12 | Saline at P1 |  |  |  |  |  | 0.005 |
|  | BIC at P1 |  |  |  |  |  |  |

| *P* value for **Fig. 4B *bottom*** | | P6 | | P9 | | P12 | |
| --- | --- | --- | --- | --- | --- | --- | --- |
|  |  | Saline at P1 | BIC at P1 | Saline at P1 | BIC at P1 | Saline at P1 | BIC at P1 |
| P6 | Saline at P1 |  | > 0.999 | 0.967 | < 0.001 | 0.011 | < 0.001 |
|  | BIC at P1 |  |  | 0.998 | < 0.001 | 0.020 | < 0.001 |
| P9 | Saline at P1 |  |  |  | < 0.001 | 0.121 | < 0.001 |
|  | BIC at P1 |  |  |  |  | < 0.001 | < 0.001 |
| P12 | Saline at P1 |  |  |  |  |  | < 0.001 |
|  | BIC at P1 |  |  |  |  |  |  |
